# Manniosides G-J, New Ursane- and Lupane-Type Saponins from *Schefflera mannii* (Hook.f.) Harms

**DOI:** 10.3390/molecules29153447

**Published:** 2024-07-23

**Authors:** Simionne Lapoupée Kuitcha Tonga, Billy Toussie Tchegnitegni, Xavier Siwe-Noundou, Ulrich Joël Tsopmene, Beaudelaire Kemvoufo Ponou, Jean Paul Dzoyem, Madan Poka, Patrick H. Demana, Léon Azefack Tapondjou, Denzil R. Beukes, Edith M. Antunes, Rémy Bertrand Teponno

**Affiliations:** 1 Research Unit of Environmental and Applied Chemistry, Faculty of Science, University of Dschang, Dschang P.O. Box 67, Cameroon; simionnekuitcha@gmail.com (S.L.K.T.); billytoussie@yahoo.fr (B.T.T.); beaudelaireponou@yahoo.fr (B.K.P.); tapondjou2001@yahoo.fr (L.A.T.); 2 School of Pharmacy, University of the Western Cape, Bellville 7535, South Africa; dbeukes@uwc.ac.za; 3 Department of Chemistry, University of the Western Cape, Bellville 7535, South Africa; 4 Department of Pharmaceutical Sciences, School of Pharmacy, Sefako Makgatho Health Sciences University, P.O. Box 218, Pretoria 0208, South Africa; madan.poka@smu.ac.za (M.P.); patrick.demana@smu.ac.za (P.H.D.); 5 Research Unit of Microbiology and Antimicrobial Substances, Faculty of Science, University of Dschang, Dschang P.O. Box 67, Cameroon; ulrichtsopmene@yahoo.com (U.J.T.); jpdzoyem@yahoo.fr (J.P.D.)

**Keywords:** *Schefflera mannii*, Araliaceae, triterpenoid saponins, structure elucidation, antibacterial activity

## Abstract

Four previously unreported triterpenoid saponins named 3β-hydroxy-23-oxours-12-en-28-oic acid 28-*O*-β-*D*-glucopyranosyl ester (mannioside G) (**1**), 23-*O*-acetyl-3β-hydroxyurs-12-en-28-oic acid 28-*O*-β-*D*-glucopyranosyl ester (mannioside H) (**2**), ursolic acid 28-*O*-[α-*L*-rhamnopyranosyl-(1→4)-β-*D*-glucopyranosyl-(1→6)-β-*D*-glucopyranosyl] ester (mannioside I) (**3**), and 3β-hydroxy-23-oxolup-20(29)-en-28-oic acid 28-*O*-β-*D*-glucopyranosyl ester (mannioside J) (**4**) were isolated as minor constituents from the EtOAc soluble fraction of the MeOH extract of the leaves of *Schefflera mannii* along with the known compounds 23-hydroxyursolic acid 28-*O*-β-*D*-glucopyranosyl ester (**5**), ursolic acid 28-*O*-β-*D*-glucopyranosyl ester (**6**), pulsatimmoside B (**7**) betulinic acid 28-*O*-[α-*L*-rhamnopyranosyl-(1→4)-β-*D*-glucopyranosyl-(1→6)-β-*D*-glucopyranosyl] ester (**8**), 23-hydroxy-3-oxo-urs-12-en-28-oic acid (**9**), hederagenin (**10**), ursolic acid (**11**), betulinic acid (**12**), and lupeol (**13**). Their structures were elucidated by a combination of 1D and 2D NMR analysis and mass spectrometry. The MeOH extract, the EtOAc and *n*-BuOH fractions, and some of the isolated compounds were evaluated for their antibacterial activity against four bacteria: *Staphylococcus aureus* ATCC1026, *Staphylococcus epidermidis* ATCC 35984, *Escherichia coli* ATCC10536, and *Klepsiella pnemoniae* ATCC13882. They were also screened for their antioxidant properties, but no significant results were obtained.

## 1. Introduction

*Schefflera mannii* (Hook.f.) Harms, also recognized as *Astropanax mannii*, is a member of the Araliaceae family, which contains 602 known species indigenous to Asia, Africa, and the southwest Pacific regions [1,2]. Plants of the genus *Schefflera* are used in traditional medicine to treat various ailments, including inflammation, rheumatism, fever, pain, diarrhea, cancer, traumatic pain, liver diseases, wound healing, chronic cough, malaria, and bites from animals and as a general tonic [2]. Although sesquiterpenes, phenylpropanoids, and lignans are obtained from some *Schefflera* species, the most common group of secondary metabolites extracted from this genus are triterpenoids and their glycosides [3,4,5,6]. Triterpene glycosides, also called saponins, are reported to exhibit a large spectrum of biological activities, including anti-inflammatory [7,8], antibacterial [9,10], antiviral [11,12], cytotoxic [13], and antioxidant [14,15] activities. Recently, we described the isolation and structure elucidation of fifteen saponins, including five new derivatives from the MeOH extract of *Schefflera mannii* [3]. In this paper, the EtOAc soluble fraction of the MeOH extract of this plant was investigated, leading to the discovery of thirteen secondary metabolites among with four previously unreported ursane and lupane glycosides.

## 2. Results and Discussion

**Figure 1:**
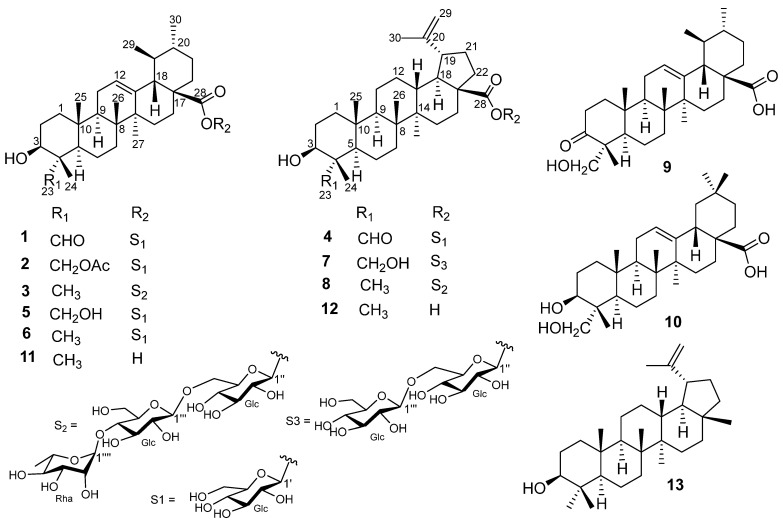
Structures of compounds **1**–**13** isolated from *S. mannii.*

Chemical examination of the EtOAc soluble fraction of the MeOH extract of *S. mannii* led to the isolation and structure elucidation of thirteen secondary metabolites, including four previously undescribed triterpene saponins, namely manniosides G–J (**1**–**4**). The known compounds were identified to be 3β,23-dihydroxyurs-12-en-28-oic acid 28-*O*-β-*D*-glucopyranosyl ester (**5**) [16], 3β-hydroxyurs-12-en-28-oic acid 28-*O*-β-*D*-glucopyranosyl ester (**6**) [17,18], 3β,23-dihydroxy-lup-20(29)-en-28-oic acid 28-*O*-[β-*D*-glucopyranosyl(1→6)-β-*D*-glucopyranosyl] ester (pulsatimmoside B) (**7**) [19], 3-hydroxylup-20(29)-en-28-oic acid 28-*O*-[α-L-rhamnopyranosyl-(1→4)-β-*D*-glucopyranosyl-(1→6)-β-*D*-glucopyranosyl] ester (**8**) [20], 23-hydroxy-3-oxo-urs-12-en-28-oic acid (**9**) [21], hederagenin (**10**) [22], ursolic acid (**11**) [23], betulinic acid (**12**) [23], and lupeol (**13**) [24] (Figure 1).

The HRESIMS of compound **1** obtained as a white gum displayed the sodium adduct ion peak at *m*/*z* 655.3816 [M + Na]^+^ corresponding to the molecular formula C_36_H_56_O_9_ (Calcd. for C_36_H_56_NaO_9_+: 655.3817). Its IR spectrum displayed characteristic absorption for hydroxyl groups (3362 cm^−^^1^), carbonyl functionalities (1728 cm^−^^1^), ethylenic double bond (1641 cm^−^^1^), and glycosidic linkage bands (1045 cm^−^^1^) [25,26].

**Figure 2:**
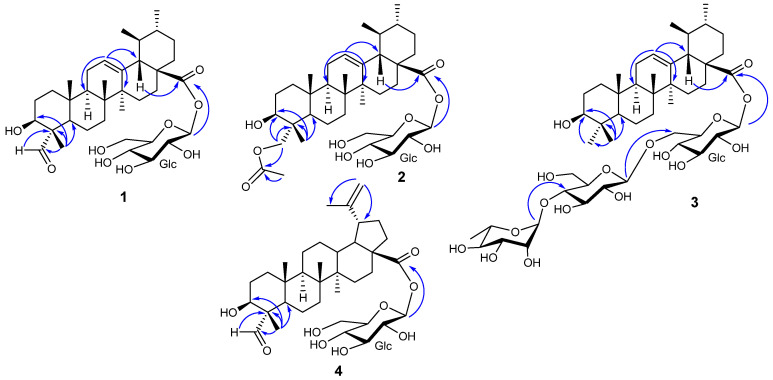
Selected HMBC correlations for compounds **1**–**4.**

The ^1^H NMR spectrum of compound **1** exhibited six methyl signals (Table 1 and Table 2), including four singlets at *δ*_H_ 0.90 (CH_3_-24, CH_3_-25), 0.74 (CH_3_-26), and 1.07 (CH_3_-27) as well as two doublets at *δ*_H_ 0.80 (*J* = 6.5 Hz, CH_3_-29) and 0.86 (*J* = 5.8 Hz, CH_3_-30). It also showed one olefinic proton signal at *δ*_H_ 5.16 (brt, *J* = 3.8 Hz, H-12), one oxygen-bearing methine proton at *δ*_H_ 3.67 (m, H-3), as well as a formyl proton resonance at *δ*_H_ 9.20 (s, H-23). The signal of one anomeric proton at *δ*_H_ 5.24 (d, *J* = 8.0 Hz, H-1′) linked to the carbon at *δ*_C_ 94.3 (C-1′) was also found. Careful analysis of the HSQC and HMBC spectra revealed the presence of 36 carbon signals, including those of one aldehyde carbonyl at *δ*_C_ 207.1 (C-23), two olefinic carbons at *δ*_C_ 125.6 (C-12) and 138.0 (C-13), one ester carbonyl at *δ*_C_ 176.6 (C-28), and one anomeric carbon at *δ*_C_ 94.3 (C-1′). Some other important signals were those observed at *δ*_C_ 71.4 (C-3), 72.5 (C-2′), 77.3 (C-3′), 69.7 (C-4′), 76.5 (C-5′), and 61.0 (C-6′). Examination of the ^1^H-^1^H COSY, HSQC, and HMBC spectra (Supplementary Materials) allowed the assignment of all the signals belonging to the aglycone moiety identified as 3β-hydroxy-23-oxours-12-en-28-oic acid. Some important HMBC correlations were observed from CH_3_-24 (*δ*_H_ 0.90, s) to C-3 (*δ*_C_ 71.4), C-4 (*δ*_C_ 55.4), C-5 (*δ*_C_ 47.3), and C-23 (*δ*_C_ 207.1) as well as from the aldehydic proton H-23 (*δ*_H_ 9.20, s) to C-4 (*δ*_C_ 55.4) (Figure 2). The β orientation of the hydroxyl group at C-3 was supported by the ROESY correlations between H-23 (*δ*_H_ 9.20, s) and H-5 (*δ*_H_ 1.22, o) and between H-23 (*δ*_H_ 9.20, s) and H-3 (*δ*_H_ 3.67, o). Comparison of 1D and 2D NMR data of the sugar residue with those reported in the literature [27] provided evidence that the residue was a glucopyranosyl unit. Furthermore, the coupling constant observed for the anomeric proton (*J* = 8.0 Hz) indicated that this glucopyranosyl unit was in the β configuration. The *D* configuration was assumed, as it is most commonly encountered in the plant kingdom [14,28]. This was supported by the fact that more than 200 triterpenoid saponins have been isolated from plants of the genus *Schefflera*, and in their structures, all the glucopyranosyl units are of *D* configuration [5,29,30,31,32]. Furthermore, fifteen triterpene saponins were recently isolated from *Schefflera mannii*, and the configuration of their glucopyranosyl units was determined to be D by GC analysis [3]. In the HMBC spectrum, the correlation depicted from the anomeric proton at *δ*_H_ 5.24 (d, *J* = 8.0 Hz, H-1′) to the carbon at *δ*_C_ 176.6 (C-28) revealed that the glucopyranosyl unit was linked at C-28. Based on the above corroboration, the structure of compound **1** was elucidated as 3β-hydroxy-23-oxours-12-en-28-oic acid 28-*O*-β-*D*-glucopyranosyl ester, a previously unreported saponin to which the trivial name mannioside G was given.

The positive ion mode HRESIMS of compound **2**, also isolated as a white gum, showed the sodium adduct ion peak at *m/z* 699.4080 [M + Na]^+^ corresponding to the molecular formula C_38_H_60_O_10_ (Calcd. for C_38_H_60_O_10_Na^+^: 699.4079). The strong IR absorption bands observed at 3354 and 1045 cm^−1^ supported its glycosidic nature. The NMR resonances (Table 1 and Table 2) of an olefin proton at *δ*_H_ 5.27 (brt, *J* = 3.8, H-12) and the methyl protons at *δ*_H_ 0.76 (s, H-24), 1.02 (s, H-25), 0.86 (s, H-26), 1.12 (s, H-27), *δ*_H_ 0.92 (d, *J* = 6.4, H-29), and 0.99 (o, H-30) as well as that of the hydroxy methine proton at *δ*_H_ 3.55 (dd, *J* = 11.4, 5.1 Hz, H-29), giving HSQC correlations with carbons at *δ*_C_ 125.8 (C-12), 11.4 (C-24), 15.1 (C-25), 16.6 (C-26), 22.5 (C-27), 16.3 (C-29), 20.1 (C-30), and 71.3 (C-3), respectively, together with the ester carbonyl signal at *δ*_C_ 176.5 (C-28) indicated that compound **2** was also an ursane derivative.

Comparison of the aglycone portion of compound **2** with that of **1** (Table 1 and Table 2) revealed that the formyl group present in compound **1** was replaced by an *O*-acetylated oxymethylene group. This was further supported by the methyl singlet observed at *δ*_H_ 2.07 (CH_3_CO) and the HMBC correlations depicted from CH_3_-24 (*δ*_H_ 0.76, s) to C-3 (*δ*_C_ 71.3), C-4 (*δ*_C_ 41.4), C-5 (*δ*_C_ 47.5), and C-23 (*δ*_C_ 65.6) and from H-23a (*δ*_H_ 4.03, d, *J* = 11.3) and H-23b (*δ*_H_ 3.90, d, *J* = 11.3) to CH_3_CO (*δ*_C_ 171.4), C-3 (*δ*_C_ 71.3), and C-5 (*δ*_C_ 47.5). Full assignment of all the proton and carbon signals of the aglycone of compound **2** was accomplished by careful examination of the HSQC, HMBC, and ^1^H-^1^H COSY spectra, which was finally elucidated as 23-*O*-acetyl-3β-hydroxyurs-12-en-28-oic acid. In the ROESY spectrum, the correlation depicted between H-3 (*δ*_H_ 3.55, dd, *J* = 11.4, 5.1 Hz) and one of the hydroxymethylene protons at *δ*_H_ 3.90 (d, *J* = 11,3 Hz, H-23b) supported the β orientation of the hydroxyl group at C-3. The sugar residue was shown to be constituted of a glucopyranosyl unit following careful examination of the 1D and 2D NMR data (Supplementary Materials) in comparison with those reported in the literature [27]. Its *β* configuration was deduced from the large coupling constant observed between H-1′ and H-2′ (*J* = 8.1 Hz), while the *D* configuration was assumed to be the same as for compound **1**. The HMBC correlation observed from H-1′ (*δ*_H_ 5.36, d, *J* = 8.0 Hz) to the carbon at *δ*_C_ 176.5 (C-28) supported the attachment of the sugar unit at C-28. Accordingly, the structure of compound **2** was determined to be 23-*O*-acetyl-3β-hydroxyurs-12-en-28-oic acid 28-*O*-β-*D*-glucopyranosyl ester, a new ursane-type saponin to which the trivial name mannioside H was assigned.

The molecular formula of compound **3** was deduced to be C_48_H_78_O_17_ from the HRESIMS data, which displayed the sodium adduct ion peak at *m/z* 949.5143 [M + Na]^+^ (Calcd. for C_48_H_78_O_17_Na^+^: 949.5131). Its IR spectrum exhibited absorption bands characteristic of hydroxyl groups (3346 cm^−1^_)_, carbonyl (1729 cm^−1^), C=C double bond (1644 cm^−1^), and glycosidic linkages (1040 cm^−1^). The ^1^H NMR spectrum of this compound showed in addition to methyl resonances (Table 1 and Table 2) at *δ*_H_ 0.99 (o, CH_3_-23/CH_3_-30), 0.79 (s, CH_3_-4), 0.98 (o, CH_3_-25) 0.85 (s, CH_3_-26), 1.12 (s, CH_3_-27), 0.92 (d, *J* = 6.5 Hz, CH_3_-29), and 1.29 (d, *J* = 6.2 Hz, CH_3_-6‴) signals ascribed to three anomeric protons at *δ*_H_ 5.32 (d, *J* = 8.1 Hz, H-1′), 4.40 (d, *J* = 7.9 Hz, H-1”), and 4.86 (o, H-1‴), giving HSQC correlations to carbons at *δ*_C_ 94.6 (C-1′), 103.1 (C-1″), and 101.5 (C-1‴), respectively. The signal of an olefinic proton was also observed at *δ*_H_ 5.25 (m, H-12). From careful inspection of its ^1^H, ^1^H-^1^H COSY, HSQC, and HMBC spectra, the aglycone part was identified to be 3β-hydroxyurs-12-en-28-oic acid (ursolic acid) [3]. For the sugar moiety, the chemical shifts of all the protons and carbons were determined from a combination of ^1^H-^1^H COSY, HSQC, and HMBC spectra (Supplementary Materials), starting from the anomeric protons. This permitted the identification of two glucopyranosyl units and one rhamnopyranosyl unit. The coupling constants of the anomeric protons suggested the β configuration for glucose and α for the rhamnose. The sequence and linkage sites were determined by the HMBC spectrum, in which correlations were depicted from H-1‴ (*δ*_H_ 4.86, o) to C-4” (δ_C_ 78.1), from H-1” (*δ*_H_ 4.40, d, *J* = 7.9 Hz) to C-6′ (*δ*_C_ 68.5), and from H-1′ (*δ*_H_ 5.32, d, *J* = 8.1 Hz) to C-28 (δ_C_ 176.5). Recently, the sequence (*α*-rhamnopyranosyl-(1→4)-β-glucopyranosyl-(1→6)-β-glucopyranosyl) was found in manniosides B, C, and E isolated from *S. mannii*, and the configurations of the sugars were determined to be *D* for glucose and *L* for rhamnose by GC analysis [3]. This sugar sequence was also reported in other saponins harbored in *Schefflera* species including *S. abyssinica* [4], *S. octophylla* [33], and *S. heptaphylla* [34]. Consequently, compound **3** was elucidated to be ursolic acid 28-*O*-[*α*-*L*-rhamnopyranosyl-(1→4)-β-*D*-glucopyranosyl-(1→6)-β-*D*-glucopyranosyl] ester, a new ursane-type glycoside named mannioside I.

Compound **4** was obtained as a gum with a molecular formula of C_36_H_56_O_9_ on the basis of the HRESIMS spectrum, which displayed the sodium adducts at *m*/*z* 655.3826 [M + Na]^+^ (Calcd. for C_36_H_56_O_9_Na^+^: 655.3817) and 1287.7780 [2M + Na]^+^ (Calcd. for C_72_H_112_O_18_Na^+^: 1287.7741). The IR spectrum showed absorption bands typical for hydroxy groups (3361 cm^−1^), a carbonyl group (1729 cm^−1^), a double bond (1642 cm^−1^) and glycosidic linkages (1038 cm^−1^). Its ^1^H NMR spectrum exhibited signals (Table 1 and Table 2) of methyl protons singlets at *δ*_H_ 0.88, (CH_3_-24), 0.80 (CH_3_-25), 0.86 (CH_3_-26), 0.92 (CH_3_-27), and 1.60 (CH_3_-30) as well as those of two methylenic olefin protons at *δ*_H_ 4.62 (brd, *J* = 2.4, H-29a) and 4.50 (brdd*,* J = 2.4, 1.4, H-29b). The resonances of a formyl proton and an anomeric proton were depicted at *δ*_H_ 9.17 (s, H-23) and 5.39 (d, *J* = 8.2, Hz, H-1′), respectively. Careful analysis of the HSQC and HMBC spectra evidenced a total of thirty-six carbons signals, including one ester carbonyl at *δ_C_* 174.7 (C-28), one formyl carbonyl at *δ_C_* 207.2 (C-23), an anomeric carbon *δ_C_* 93.8 (C-1′), and two olefinic carbons at *δ_C_* 150.4 (C-20) and 108.9 (C-17), suggesting that compound **4** was a lupane-type glycoside [3]. The HMBC correlations observed from the proton at *δ*_H_ 9.17 (s, H-23) to the carbon at *δ*_C_ 55.5 (C-4) and from the protons at *δ*_H_ 0.88 (s, CH_3_-24) to the carbons at *δ*_C_ 71.4 (C-3), 207.2 (C-23), and 55.5 (C-4) (Figure 2) evidenced the presence of the hydroxyl group on C-3 and that C-23 was a formyl group. The ROESY correlations observed between H-23 (*δ*_H_ 9.17, s) and H-5 (*δ*_H_ 1.19, o) and between H-23 (*δ*_H_ 9.17, s) and H-3 (*δ*_H_ 3.64, m) supported the β orientation of the hydroxyl group at C-3. The aglycone portion was then elucidated as 3*β*-hydroxy-23-oxolup-20(29)-en-28-oic acid [3]. In the ^13^C NMR spectrum (Supplementary Materials), the resonances depicted at *δ*_C_ 93.8 (C-1′), 77.4 (C-3′), 77.0 (C-5′), 72.7 (C-2′), 69.7 (C-4′), and 60.9 (C-6′) were ascribed to the sugar moiety, which was identified as a glucopyranosyl from comparison with the literature data [27]. The coupling constant of the anomeric proton (*J* = 8.2 Hz) was in favor of a *β* configuration, and the *D* configuration was assumed as that for compounds **1** and **2**. The HMBC correlation from the anomeric proton at *δ*_H_ 5.39 (d, *J* = 8.2, H-1′) and the ester carbonyl at *δ_C_* 174.7 (C-28) evidenced the linkage of the glucopyranosyl unit at C-28. Therefore, compound **4** was elucidated as a previously undescribed lupane triterpenoid saponin trivially named mannioside J.

The antibacterial activity of the crude extract, fractions, and some isolated compounds was evaluated using the broth microdilution method on two Gram-positive, namely *Staphylococcus aureus* ATCC1026 and *Staphylococcus epidermidis* ATCC35984, and two Gram-negative, namely *Escherichia coli* ATCC10536 and *Klepsiella pnemoniae* ATCC13882, bacterial strains. The results were discussed according to the antimicrobial cut-off points of plant extracts and pure compounds described by Kuete in 2010 [35]. The extract and fractions displayed inhibitory potential ranging from weak to significant (64–1024 µg/mL) against the bacterial strains tested (Table 3). The extract and all the fractions tested had moderate activity against the two Gram-negative bacteria: *Escherichia coli* ATCC10536 and *Klepsiella pnemoniae* ATCC13882 (126 < MIC < 512 µg/mL). The *n*-BuOH fraction significantly inhibited the growth of *Staphylococcus epidermidis* ATCC 35984 with an MIC value of 64 µg/mL, while the EtOAc fraction exhibited a moderate activity against *Staphylococcus aureus* ATCC1026 with an MIC value of 512 µg/mL. Compounds **5**, **9**, and **10**–**13** were not active against all the tested bacterial strains. Our results agreed with the literature on the antibacterial activity of lupeol [36] and betulinic acid [37], which were not active against some bacterial strains. However, oleanolic acid was reported to exhibit good to moderate activity against *Staphylococcus aureus* and *Escherichia coli* [37].

Since triterpenoids and their glycosides were reported to exhibit antioxidant activity [14,38], the extract, fractions, and some isolated compounds were screened for their antioxidant properties using the DPPH and FRAP methods, but no significant effect was observed when compared to l-ascorbic acid used as positive control.

## 3. Materials and Methods

### 3.1. General Experimental Procedures

IR spectra were obtained using a Perkin Elmer Spectrum 400 FT-IR/FT-NIR spectrometer (Bruker, Billerica, MA, USA), equipped with a universal ATR sampling accessory. ^1^H and ^13^C NMR spectra were recorded in CD_3_OD on a Bruker Avance 400 (400 MHz for ^1^H and 100 MHz for ^13^C) (Bruker, Ettlingen, Germany). All chemical shifts (*δ*) are given in ppm with reference to the residual solvent signal, and coupling constants (*J*) are in Hz. HRESIMS (high-resolution electrospray ionization) was recorded at the Central Analytical Facility at Stellenbosch University using a Waters Synapt G2 spectrometer (Milford, MA, USA). The ionization source was an ESI^+^, with a cone voltage 15 V. Semi-preparative HPLC (Luna 10 μm C18 (2) column) was performed with an Agilent Technologies 1260 Infinity (G4286B, 1220 LC system) HPLC pump connected to a G1362A Agilent Refractive Index Detector. The flow rate was 3 mL/min, and 250 μL of solution was injected each time. Column chromatography was performed using silica gel 60 (Merck, Darmstadt/Germany) (0.063–0.200 mm and 0.04–0.063 mm) and Sephadex LH-20. The following solvent systems were used: MeOH for Sephadex column chromatography and mixtures of hexane−EtOAc, EtOAc−MeOH, and EtOAc−MeOH−H_2_O for silica gel column chromatography. Thin-layer chromatography (TLC) was performed on Merck precoated silica gel 60 RP-18 F254S and silica gel 60 F254 aluminum foil. The plates were revealed using a UV lamp (254–365 nm) and 10% H_2_SO_4_ reagent followed by heating.

### 3.2. Plant Material

The leaves of *Schefflera mannii* (Hook.f.) Harms were collected in Dschang (5°27′0″ N and 10°4′0″ E), West Region of Cameroon, in November 2017. The plant material was identified at the Cameroon National Herbarium in Yaoundé by Mr. Nana Victor in comparison with a voucher specimen deposited under the reference N° 35063/HNC.

### 3.3. Extraction and Isolation

The air-dried and pulverized leaves (3 kg) were macerated three times with MeOH (95%) (15 L) at room temperature (each time for 24 h). The filtrate obtained was concentrated under reduced pressure to give 405 g of extract (yield 13.5%). An amount of 369 g of the crude methanolic extract was suspended in water (1 L) and successively partitioned with EtOAc (3 × 1 L) and *n*-BuOH (3 × 1 L). The solutions were evaporated under reduced pressure to afford 201.8 and 36.6 g of EtOAc and *n*-BuOH fractions, respectively. A part of the EtOAc fraction 196 g was subjected to silica gel column chromatography eluted with hexane–EtOAc (from hexane–EtOAc 10% to EtOAc 100%) and then EtOAc–MeOH (from EtOAc 100% to EtOAc–MeOH 30%) with increasing polarity to give four sub-fractions (A–D).

Compound **9** (700 mg) was obtained from sub-fraction B (14.2 g) by recrystallization in MeOH. Sub-fraction C (43.5 g) was chromatographed on silica gel column using hex–AcOEt 40% as the eluent to afford three sub-fractions (FC-1–FC-3). The filtration of sub-fraction FC-2 (20.5 g) after crystallization in EtOAc yielded compound **10** (1.7 g). Silica gel column chromatography of sub-fraction A (3.4 g) eluted with hexane–EtOAc (from 10 to 20%) afforded five main sub-fractions (FA-1–FA-5). Further silica gel column chromatography of sub-fraction FA-5 (0.4 g) using hexane–EtOAc 15% to 25% as eluent afforded compound **11** (150 mg), while betulinic acid (**12**) (50 mg) and lupeol (**13**) (20 mg) crystallized in sub-fractions FA-3 (0.2 g) and FA-2 (0.6 g), respectively.

Sub-fraction D (85.8 g) was subjected to silica gel column chromatography using EtOAc and then EtOAc–MeOH–H_2_O 95-5-2 as mobile phase to give four sub-fractions (FD-1–FD-4). FD-2 (20.8 g) was further chromatographed on silica gel column chromatography eluted with CH_2_Cl_2_–MeOH 5% to CH_2_Cl_2_–MeOH 15% to afford sub-fractions FD-2-1, FD-2-2, FD-2-3, and FD-2-4. The filtration of sub-fraction FD-2-4 (10 g) yielded a mixture (12.2 mg), which was purified by RP-18 semi-preparative HPLC eluted with MeCN/H_2_O/FA (3:7:0.02, 3 mL/min) to afford compounds **4** (1.4 mg, *t_R_*: 31.41 min) and **1** (1.2 mg, *t_R_*: 33.13 min). Part of sub-fraction FD-2-3 (35.1 mg) was separated by RP-18 semi-preparative HPLC eluted with MeCN/H_2_O/FA (13:7:0.02, 3 mL/min) to yield compounds **2** (1.4 mg, *t_R_*: 10.21 min) and **6** (2.37 mg, *t_R_*: 14.22 min). The sub-fraction FD-3 (10.5 g) was purified on silica gel column chromatography eluted with CH_2_Cl_2_–MeOH 5% to give compound **5** (500 mg). Column chromatography of the sub-fraction FD-4 (15.7 g) using CH_2_Cl_2_–MeOH 10% followed by repeated column chromatography on Sephadex LH-20 using MeOH as mobile phase yielded a mixture (7.8 mg) that was further purified by semi-preparative RP-18 HPLC (MeCN/H_2_O/FA (3:2:0.02, 3 mL/min)) and afforded compounds **7** (2.1 mg, *t_R_*: 8.73 min), **3** (1.1 mg, *t_R_*: 10.94 min), and **8** (1.6 mg, *t_R_*: 11.80 min).

*Mannioside G (**1**)*: White gum; IR (KBr) 3362, 2939, 1728, 1641, 1452, 1378, 1045 cm^−1^; ^1^H and ^13^C NMR data, see Table 1 and Table 2; HRESI−MS (positive mode) *m/z* 655.3816 [M + Na]^+^ (Calcd. for C_36_H_56_NaO_9_^+^: 655.3817), 453.3367 [M + H-162-H_2_O]^+^.

*Mannioside H (**2**)*: White gum; IR (KBr) 3354, 2940, 1734, 1642, 1451, 1377, 1045 cm^−1^; ^1^H and ^13^C NMR data, see Table 1 and Table 2; HRESI−MS (positive mode) *m/z* 699.4080 [M + Na]^+^ (Calcd. for C_38_H_60_O_10_Na^+^: 699.4079), 1375.8317 [2M + Na]^+^.

*Mannioside I (**3**)*: White gum; IR (KBr) 3346, 2927, 1729, 1644, 1447, 1390, 1040 cm^−1^; ^1^H and ^13^C NMR data, see Table 1 and Table 2; HRESI−MS (positive mode) *m/z* 949.5143 [M + Na]^+^ (Calcd. for C_48_H_78_O_17_Na+: 949.5131).

*Mannioside J (**4**)*: White gum; IR (KBr) 3361, 2926, 1729, 1642, 1452, 1377, 1038 cm^−1^; ^1^H and ^13^C NMR data, see Table 1 and Table 2; HRESI−MS (positive mode) *m/z* 655.3826 [M + Na]^+^ (Calcd. for C_36_H_56_O_9_Na^+^: 655.3817), 1287.7780 [2M + Na]^+^.

### 3.4. The Antibacterial Activity

The antibacterial activity was conducted according to the technique described by Mbaveng et al. [39]. The microorganisms comprised two Gram-positive, namely *Staphylococcus aureus* ATCC1026 and *Staphylococcus epidermidis* ATCC35984, and two Gram-negative, namely *Escherichia coli* ATCC10536 and *Klepsiella pnemoniae* ATCC13882, bacterial strains. They were obtained from the Research Unit of Bacteriology of “Centre Pasteur de Yaoundé” (Cameroon). They were kept in the laboratory in the mixture of glycerol and Mueller Hinton Broth (MHB) (1:1) at the temperature of −4 °C, and the activation was achieved using the streak technique on agar medium. Doxycycline was used as positive control.

### 3.5. The Antioxidant Activity

The assay was carried out using the method previously described by Mensor et al. [40]. A volume of 20 µL methanol was poured in the last seven lines of a 96-well plate, and then, 20 µL of methanolic solution of samples at 2 mg/mL were poured into the first two wells of each row (four rows per sample). The dilution was conducted following a geometric series with the common ratio 2 in the other wells. After that, 180 µL of methanolic solution of DPPH (0.08 mg/mL) was put into each well of the first three rows, and the same volume of methanol was put into the well of the fourth row. The next step was the incubation of the plates for 30 min in obscurity at room temperature; and finally, the absorbance of each well was read using the spectrophotometer (FLUOstar Omega for microplate, Gainesville, FL, USA) at 517 nm and then converted into percentages of antioxidant activity. The positive control used was l-ascorbic acid. The experiment was conducted three times successively. The percentages of antioxidant activity were calculated by using the following formula.

% antioxidant activity = (⦋Abs (DPPH) − (Abs (trial) − Abs (sample)⦌)/(Abs (DPPH)) × 100

Abs (DPPH) = Absorbance of a methanolic solution of DPPH
Abs (trial) = Absorbance of a methanolic solution of DPPH + sample
Abs (sample) = Absorbance of a methanolic solution of sample

The reducing power of the tested samples was determined following the method reported by Benzie and Strain [41]. For the preparation of the FRAP reagent, a buffer solution of sodium acetate (300 mM, pH 3.6), a solution of 2,4,6-tris (2-pyridyl)-1,3,5-s-triazine TPTZ (10 mM), and a ferric chloride solution were mixed together at the ratio 10:1:1, respectively. A volume of 5 µL of each sample at 2 mg/mL was mixed with 95 µL of the FRAP reagent, and the mixture was incubated in the dark for 30 min at 37 °C. The optical density was read by using a spectrophotometer at 593 nm. l-ascorbic acid was used as positive control. The antioxidant potential of each sample was calculated from the calibration curve of ferrous sulfate solution and expressed in millimole equivalent of FeSO_4_ per gram of sample.

## 4. Conclusions

Phytochemical investigation of the EtOAc soluble fraction of the MeOH extract of the leaves of *Schefflera mannii* led to the discovery of four previously unreported triterpenoid saponins, namely manniosides G–J along, with nine known metabolites. Only very little amounts of the new compounds were obtained (less than 1.5 mg); consequently, they were not screened for their biological activities. Although compounds **5**, **9**, and **10**–**13** did not show antibacterial and antioxidant activities, the present work once again strengthens the chemotaxonomy of plants of the genus *Schefflera* since they are known to be an important source of triterpenoids and their glycosides (saponins). However, given that saponins are well known for their cytotoxic, antifungal, and anti-inflammatory properties, we intend to reisolate these compounds in large amounts in order to evaluate these biological activities during our future investigations.

## Figures and Tables

**Table 1:** ^13^C and ^1^H NMR spectroscopic data for the aglycones of compounds **1**–**4** (100 and 400 MHz resp.; CD_3_OD; δ in ppm).

Position	1		2		3		4	
	*δ*_C_	*δ*_H_ (m, *J* in Hz)	*δ*_C_	*δ*_H_ (m, *J* in Hz)	*δ*_C_	*δ*_H_ (m, *J* in Hz)	*δ*_C_	*δ*_H_ (m, *J* in Hz)
1	38.2	1.64 (o), 1.04 (m)	38.3	1.70 (o), 1.02 (o)	38.6	1.67 (m), 1.01 (o)	38.2	1.67 (o), 0.93 (o)
2	25.5	1.60 (o)	25.9	1.65 (o)	26.5	1.57 (o)	25.8	1.57 (o), 0.99 (o)
3	71.4	3.67 (o)	71.3	3.55 (dd, 11.4, 5.1)	78.1	3.16 (m)	71.4	3.64 (m)
4	55.4		41.4		38.5		55.5	
5	47.3	1.22 (o)	47.5	1.13 (o)	55.3	0.76 (o)	47.4	1.19 (o)
6	20.4	0.78 (m)	17.8	1.40 (o)	18.2	1.56 (m)	20.6	0.72 (m), 1.36 (o)
7	32.2	1.45 (o),1.17 (m)	32.6	1.33 (m), 1.48 (m)	32.9	1.34 (o), 1.51 (o)	33.4	1.36 (o), 1.20 (o)
8	39.9		39.8		39.6		42.2	
9	47.5	1.58 (o)	47.8	1.57 (o)	47.6	1.56 (o)	50.5	1.36 (o)
10	35.5		36.4		36.7		38.2	
11	23.0	1.86 (m)	23.1	1.96 (o)	23.1	1.95 (o)	20.7	1.35 (o)
12	125.6	5.16 (t, 3.8)	125.8	5.27 (brt, 3.8)	125.9	5.25 (m)	25.2	1.64 (m), 0.99 (m)
13	138.0		137.8		137.9		38.1	2.20 (o)
14	41.9		41.8		41.7		41.1	
15	28.0	1.94 (m) 1.08 (m)	27.9	1.95 (o) 1.09 (o)	27.5	1.93 (o) 1.07 (o)	29.5	1.45(dd, 13.0, 3.3), 1.02 (o)
16	23.6	1.87 (m), 1.64 (o)	23.9	2.08 (o), 1.77 (m)	25.2	1.75 (o)	31.4	2.25 (o), 1.36 (o)
17	47.7		47.9		48.2		56.6	
18	52.9	2.14 (d, 11.3)	52.9	2.25 (d, 11.3)	52.8	2.25 (m)	49.2	1.57 (o)
19	38.9	1.30 (o)	30.0	1.41 (o)	39.1	1.41 (m)	47.1	2.91 (td, 11.0, 4.6)
20	38.8	0.88 (m)	38.8	0.99 (o)	39.0	0.98 (o)	150.4	
21	29.5	1.41 (o), 1.31 (o)	30.4	1.52 (o), 1.36 (m)	30.4	1.52 (o), 1.35 (o)	30.1	1.83 (o), 1.28 (o)
22	36.1	1.52 (m), 1.66 (o)	36.1	1.77 (o), 1.66 (o)	36.3	1.75 (o), 1.62 (o)	36.5	1.88 (m), 1.35 (o)
23	207.1	9.20 (s)	65.6	4.03 (d, 11.3) 3.90 (d, 11.3)	27.5	0.99 (o)	207.2	9.17 (s)
24	8.1	0.90 (o)	11.4	0.76 (s)	15.0	0.79 (s)	7.7	0.88 (s)
25	15.0	0.90 (o)	15.1	1.02 (s)	14.9	0.98 (o)	15.4	0.80 (s)
26	16.5	0.74 (s)	16.6	0.86 (s)	16.6	0.85 (s)	15.2	0.86 (s)
27	22.6	1.04 (s)	22.5	1.12 (o)	22.6	1.12 (s)	13.7	0.92 (s)
28	176.6		176.5		176.5		174.7	
29	16.3	0.80 (d, 6.5)	16.3	0.92 (d, 6.4)	16.3	0.92 (d, 6.5)	108.9	4.62 (brd, 2.4) 4.50 (brdd, 2.4, 1.4)
30	20.3	0.86 (d, 5.8)	20.1	0.99 (o)	20.2	0.99 (d, 7.2)	18.2	1.60 (s)
23-COCH_3_			171.4					
23-COCH_3_			19.4	2.07 (s)				

**Table 2:** ^13^C and ^1^H NMR spectroscopic data for the sugar units of compounds **1**–**4** (100 and 400 MHz, respectively; CD_3_OD; *δ* in ppm).

Position	1		2		3		4	
	*δ*_C_	*δ*_H_ (m, *J* in Hz)	*δ*_C_	*δ*_H_ (m, *J* in Hz)	*δ*_C_	*δ*_H_ (m, *J* in Hz)	*δ*_C_	*δ*_H_ (m, *J* in Hz)
1′	94.3	5.24 (d, 8.0)	94.4	5.36 (d, 8.1)	94.6	5.32 (d, 8.1)	93.8	5.39 (d, 8.2)
2′	72.5	3.21 (o)	72.5	3.32 (o)	72.5	3.35 (o)	72.7	3.21 (o)
3′	77.3	3.24 (o)	77.1	3.35 (o)	76.8	3.43 (o)	77.4	3.27 (o)
4′	69.7	3.25 (o)	69.8	3. 37 (o)	69.7	3.42 (0)	69.7	3.27 (o)
5′	76.5	3.28 (o)	76.9	3.41 (o)	76.5	3.51 (o)	77.0	3.27 (o)
6′	61.0	3.58 (dd, 11.9, 4.3) 3.68 (o)	61.9	3.58 (dd, 11.9, 2.0) 3.68 (dd, 11.9, 4.3)	68.5	4.10 (dd, 11.8, 2.0) 3.78 (o)	60.9	3.60 (o) 3.73 (m)
1″					103.1	4.40 (d, 7.9)		
2″					73.9	3.25 (dd, 9.0, 7.9)		
3″					75.4	3.31 (o)		
4″					78.1	3.55 (o)		
5″					75.6	3.47 (o)		
6″					60.5	3.82 (o), 3.66 (o)		
1‴					101.5	4.86 (o)		
2‴					71.0	3.85 (o)		
3‴					70.9	3.64 (o)		
4‴					72.4	3.42 (o)		
5‴					69.3	3.99 (dd, 9.6, 6.2)		
6‴					16.4	1.29 (d, 6.2)		

**Table 3:** Minimum inhibitory concentrations of the extract, fractions, and some compounds isolated from *S. mannii.*

Samples	Minimum Inhibitory Concentrations (MIC, µg/mL)			
	SA1026	SE35984	EC10536	KP13882
MeOH extract	1024	nd	512	512
EtOAc fraction	512	128	128	256
*n*-BuOH fraction	1024	64	256	256
**5**	nd	nd	nd	nd
**9**	nd	nd	nd	nd
**10**	1024	nd	nd	nd
**11**	nd	nd	nd	nd
**12**	nd	nd	nd	nd
**13**	nd	nd	nd	nd
Doxycycline	2	8	2	2

SA 1026: *Staphylococcus aureus* ATCC1026; SE35984: *Staphylococcus epidermidis* ATCC 35984; EC10536: *Escherichia coli* ATCC10536; KP13882: *Klepsiella pnemoniae* ATCC13882; nd: not determined.

## Data Availability

Data are contained within the article and Supplementary Material.

## Supplementary Materials

The following supporting information can be downloaded at: https://www.mdpi.com/article/10.3390/molecules29153447/s1, MS spectra, HRESIMS, ^1^H and ^13^C NMR, ^1^H-^1^H COSY, HSQC, and HMBC spectra are available as Supplementary Material. Figure S1. HRESIMS of compound **1**. Figure S2. IR spectrum of compound **1**. Figure S3. ^1^H NMR spectrum of compound **1**. Figure S4. ^1^H-^1^H COSY spectrum of compound **1**. Figure S5. HSQC spectrum of compound **1**. Figure S6. HMBC spectrum of compound **1**. Figure S7. ROESY spectrum of compound **1**. Figure S8. HRESIMS of compound **2**. Figure S9. IR spectrum of compound **2**. Figure S10. ^1^H NMR spectrum of compound **2**. Figure S11. ^1^H-^1^H COSY spectrum of compound **2**. Figure S12. HSQC spectrum of compound **2**. Figure S13. HMBC spectrum of compound **2**. Figure S14. ROESY spectrum of compound **2**. Figure S15. HRESIMS of compound **3**. Figure S16. IR spectrum of compound **3**. Figure S17. ^1^H NMR spectrum of compound **3**. Figure S18. ^1^H-^1^H COSY spectrum of compound **3**. Figure S19. HSQC spectrum of compound **3**. Figure S20. HMBC spectrum of compound **3**. Figure S21. HRESIMS of compound **4**. Figure S22. IR spectrum of compound **4**. Figure S23. ^1^H NMR spectrum of compound **4**. Figure S24. ^13^C NMR spectrum of compound **4**. Figure S25. ^1^H-^1^H COSY spectrum of compound **4**. Figure S26. HSQC spectrum of compound **4**. Figure S27. HMBC spectrum of compound **4**. Figure S28. ROESY spectrum of compound **4**.
