# Supplementary material for: Manniosides G-J, New Ursane- and Lupane-Type Saponins from Schefflera mannii (Hook.f.) Harms
Source: Molecules. 2024 Jul 23;29(15):3447. doi: 10.3390/molecules29153447 (PMC11313943; doi:10.3390/molecules29153447)
Supplement: Supplementary file 1 [file molecules-29-03447-s001.zip › molecules-3106570-supplementary.pdf]

Manniosides G-J, new ursane- and lupane-type saponins from *Schefflera mannii* (Hook.f.) Harms

Simionne Lapoupée Kuitcha Tonga[1], Billy Toussie Tchegnitegni[1,2,3], Xavier Siwe-Noundou[4,*], Ulrich Joël Tsopmene[5], Beaudelaire Kemvoufo Ponou[1], Jean Paul Dzoyem[5], Madan Poka[4], Patrick H. Demana[4], Léon Azefack Tapondjou[1], Denzil R. Beukes[2], Edith M. Antunes[3,*], Rémy Bertrand Teponno[1,*]

1 Research Unit of Environmental and Applied Chemistry, Faculty of Science, University of Dschang, P.O. Box 67, Dschang, Cameroon; simionnekuitcha@gmail.com, billytoussie@yahoo.fr, beaudelaireponou@yahoo.fr, tapondjou2001@yahoo.fr, remyteponno@gmail.com

2 School of Pharmacy, University of the Western Cape, Bellville 7535, South Africa; billytoussie@yahoo.fr, bbeukes@uwc.ac.za

3 Department of Chemistry, University of the Western Cape, Bellville 7535, South Africa; billytoussie@yahoo.fr, ebeukes@uwc.ac.za

4 Department of Pharmaceutical Sciences, School of Pharmacy, Sefako Makgatho Health Sciences University, P.O. Box 218, Pretoria 0208, South Africa; xavier.siwenoundou@smu.ac.za, madan.poka@smu.ac.za, patrick.demana@smu.ac.za

5 Research Unit of Microbiology and Antimicrobial Substances, Faculty of Science, University of Dschang, P.O. Box 67, Dschang, Cameroon; ulrichtsopmene@yahoo.com, jpdzoyem@yahoo.fr

* Correspondence: xavier.siwenoundou@smu.ac.za (X.S.-N.); ebeukes@uwc.ac.za (E.M.A.); remyteponno@gmail.com (R.B.T.)

Abstract: Four previously unreported triterpenoid saponins named 3β-hydroxy-23-oxours-12-en-28-oic acid 28-*O*-β-*D*-glucopyranosyl ester (Mannioside G) (**1**), 23-*O*-acetyl-3β-hydroxyurs-12-en-28-oic acid 28-*O*-β-*D*-glucopyranosyl ester (Mannioside H) (**2**), ursolic acid 28-*O*-[α-*L*-rhamnopyranosyl-(1→4)-β-*D*-glucopyranosyl-(1→6)-β-*D*-glucopyranosyl] ester (Mannioside I) (**3**), and 3β-hydroxy-23-oxolup-20(29)-en-28-oic acid 28-*O*-β-*D*-glucopyranosyl ester (Mannioside I) (**4**) were isolated from the EtOAc soluble fraction of the MeOH extract of the leaves of *Schefflera mannii* along with the known compounds 3β,23-dihydroxyurs-12-en-28-oic acid 28-*O*-β-*D*-glucopyranosyl ester (**5**), 3β-hydroxyurs-12-en-28-oic acid 28-*O*-β-*D*-glucopyranosyl ester (**6**), 3β,23-dihydroxy-lup-20(29)-en-28-oic acid 28-*O*-[β-*D*-glucopyranosyl(1→6)-β-*D*-glucopyranosyl] ester (Pulsatimmoside B) (**7**) 3-hydroxylup-20(29)-en-28-oic acid 28-*O*-[α-*L*-rhamnopyranosyl-(1→4)-β-*D*-glucopyranosyl-(1→6)-β-*D*-glucopyranosyl] ester (**8**), 23-hydroxy-3-oxo-urs-12-en-28-oic acid (**9**), hederagenin (**10**), ursolic acid (**11**), betulinic acid (**12**), and lupeol (**13**). Their structures were elucidated by a combination of 1D and 2D NMR analysis and mass spectrometry. The MeOH extract, the EtOAc and *n*-BuOH fractions, and some of the isolated compounds were evaluated for their antibacterial activity against four bacteria: *Staphylococcus aureus* ATCC1026, *Staphylococcus epidermidis* ATCC 35984, *Escherichia coli* ATCC10536, and *Klepsiella pnemoniae* ATCC13882. They were also screened for their antioxidant properties but no significant results were obtained.

Keywords: *Schefflera mannii*; Araliaceae; triterpenoid saponins; structure elucidation; antibacterial activity

Contents

Figure S1. HRESIMS of compound **1**

Figure S2. IR spectrum of compound **1**

Figure S3. ^{1}H NMR spectrum of compound **1**

Figure S4. ^{1}H-^{1}H COSY spectrum of compound **1**

Figure S5. HSQC spectrum of compound **1**

Figure S6. HMBC spectrum of compound **1**

Figure S7. ROESY spectrum of compound **1**

Figure S8. HRESIMS of compound **2**

Figure S9. IR spectrum of compound **2**

Figure S10. ^{1}H NMR spectrum of compound **2**

Figure S11. ^{1}H-^{1}H COSY spectrum of compound **2**

Figure S12. HSQC spectrum of compound **2**

Figure S13. HMBC spectrum of compound **2**

Figure S14. ROESY spectrum of compound **2**

Figure S15. HRESIMS of compound **3**

Figure S16. IR spectrum of compound **3**

Figure S17. ^{1}H NMR spectrum of compound **3**

Figure S18. ^{1}H-^{1}H COSY spectrum of compound **3**

Figure S19. HSQC spectrum of compound **3**

Figure S20. HMBC spectrum of compound **3**

Figure S21. HRESIMS of compound **4**

Figure S22. IR spectrum of compound **4**

Figure S23. ^{1}H NMR spectrum of compound **4**

Figure S24. ^{13}C NMR spectrum of compound **4**

Figure S25. ^{1}H-^{1}H COSY spectrum of compound **4**

Figure S26. HSQC spectrum of compound **4**

Figure S27. HMBC spectrum of compound **4**

Figure S28. ROESY spectrum of compound **4**

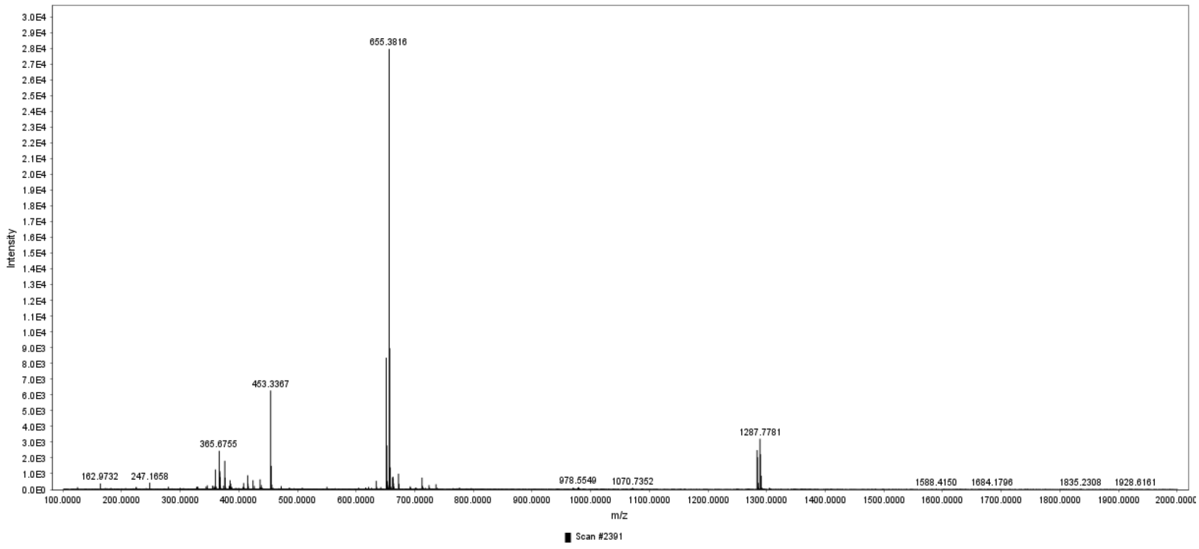

Figure S1. HRESIMS of compound **1**

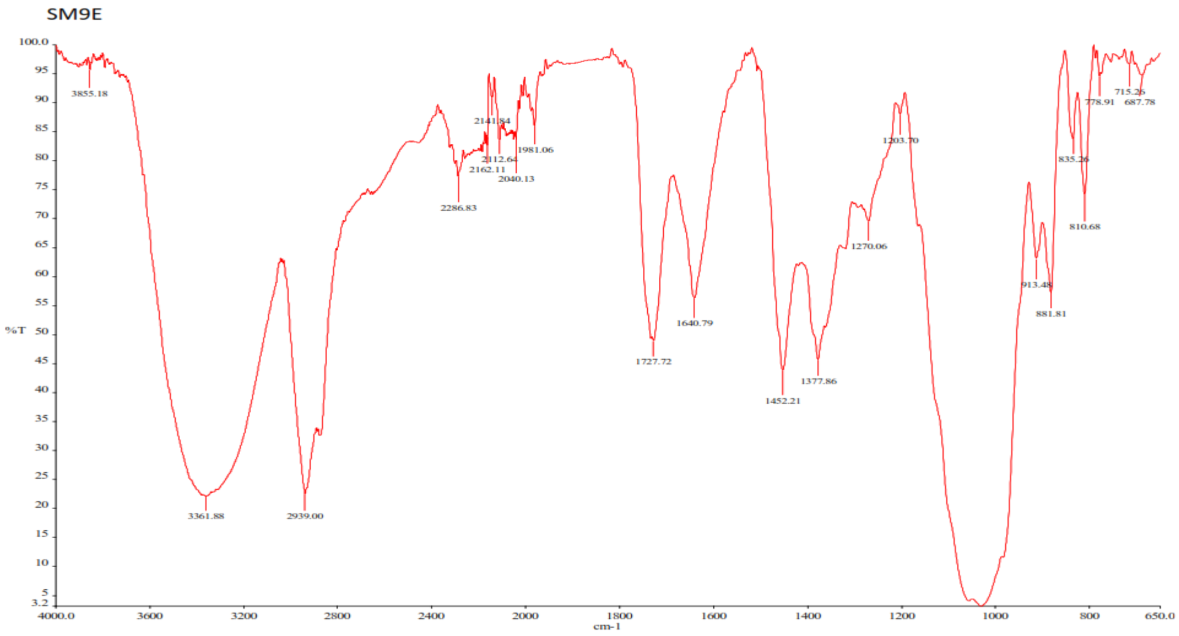

Figure S2. IR spectrum of compound **1**

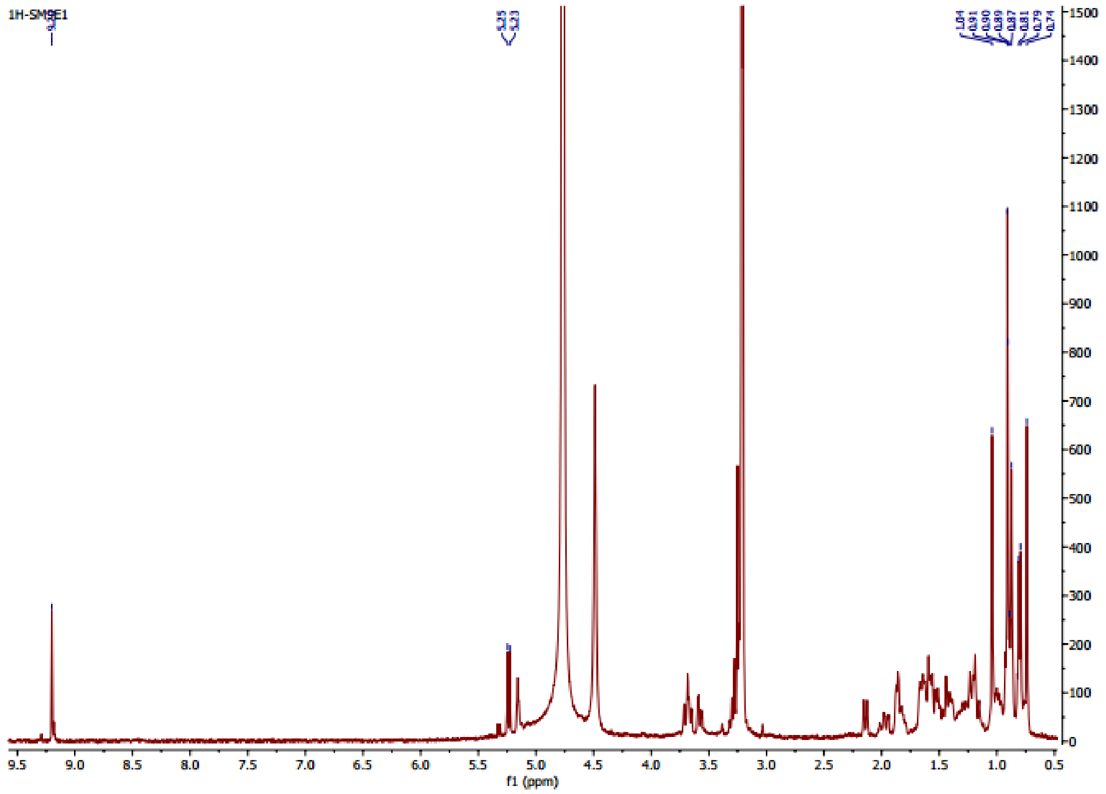

Figure S3. ^{1}H NMR spectrum of compound **1**

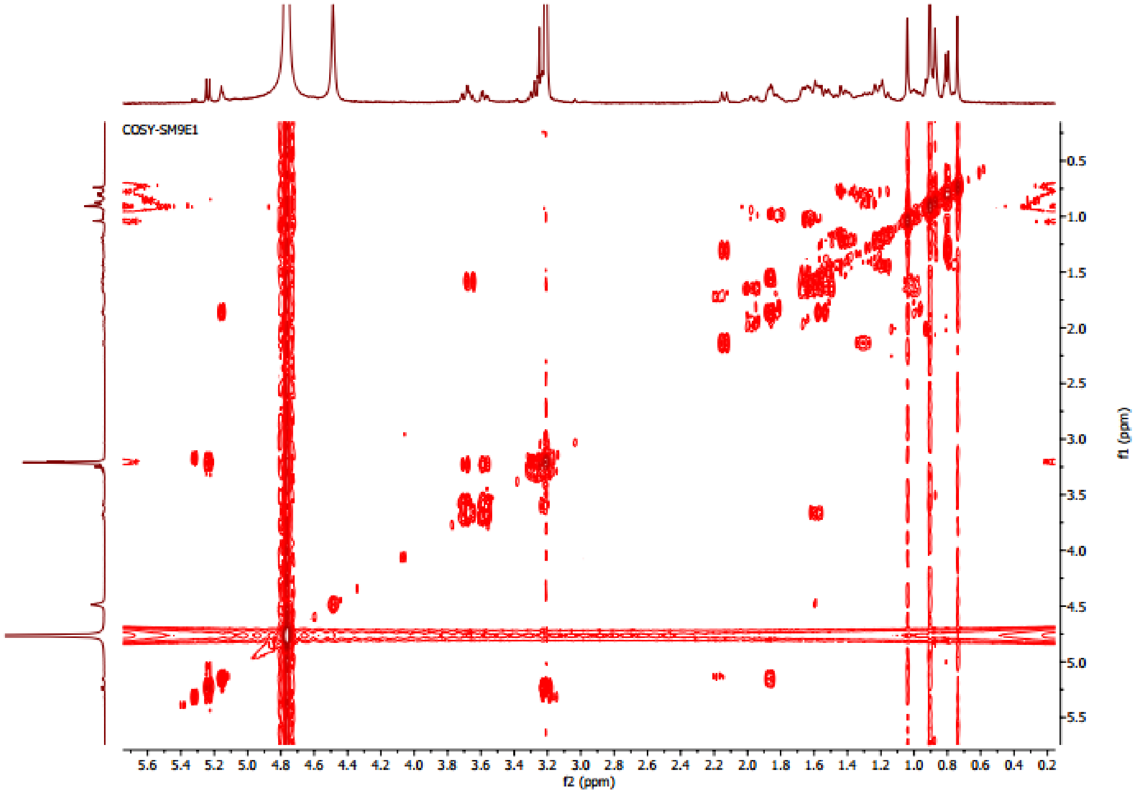

Figure S4. ^{1}H-^{1}H COSY spectrum of compound **1**

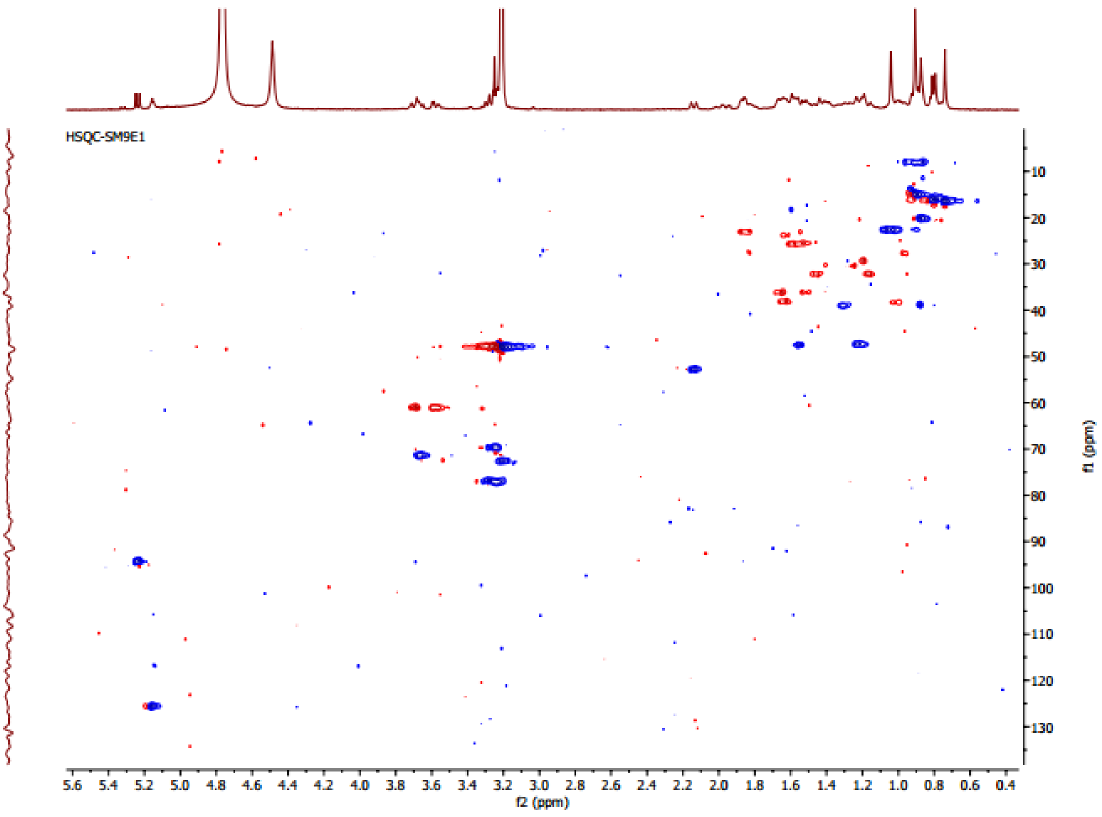

Figure S5. HSQC spectrum of compound **1**

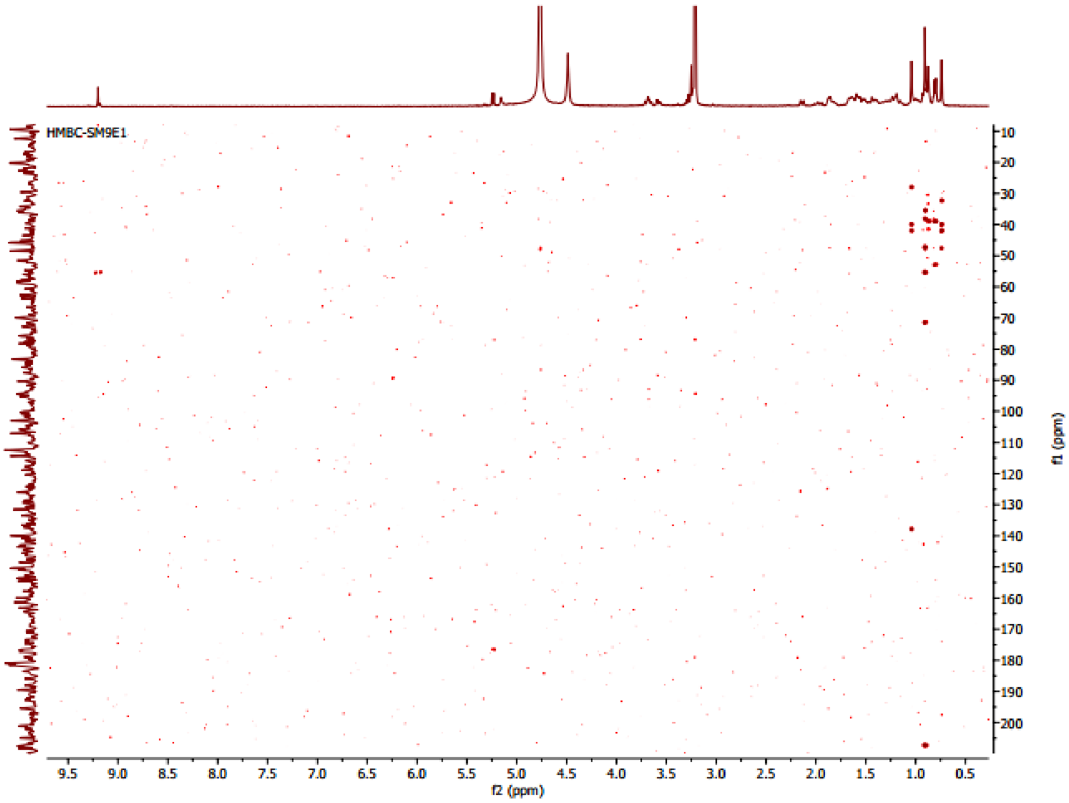

Figure S6. HMBC spectrum of compound **1**

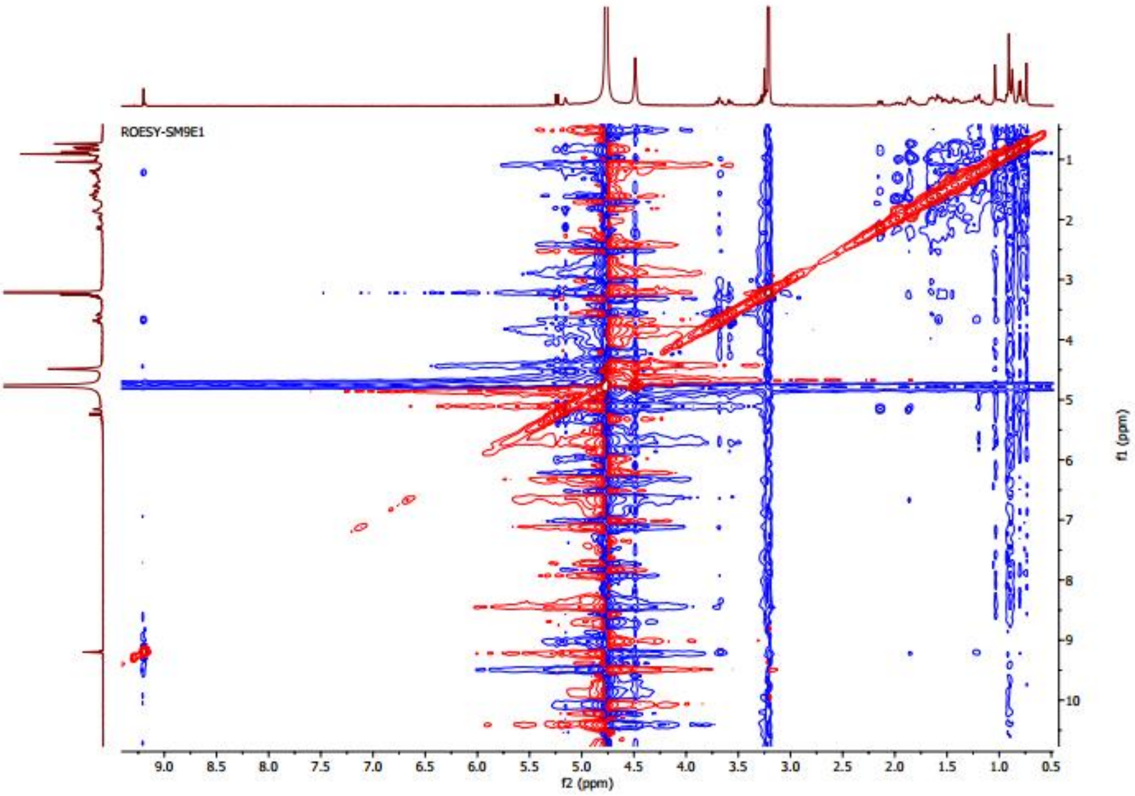

Figure S7. ROESY spectrum of compound **1**

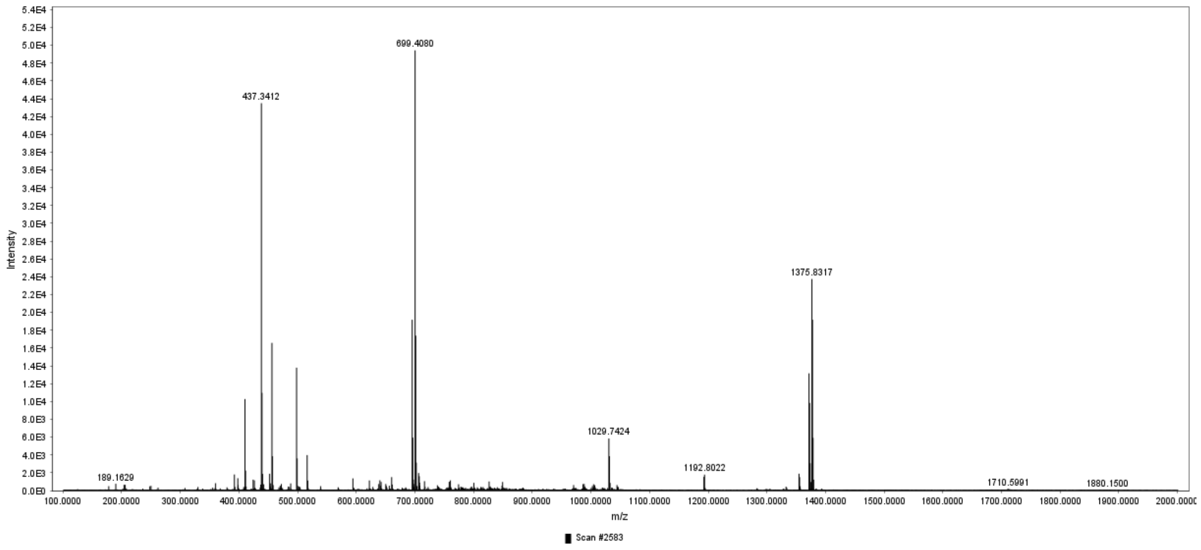

Figure S8. HRESIMS of compound **2**

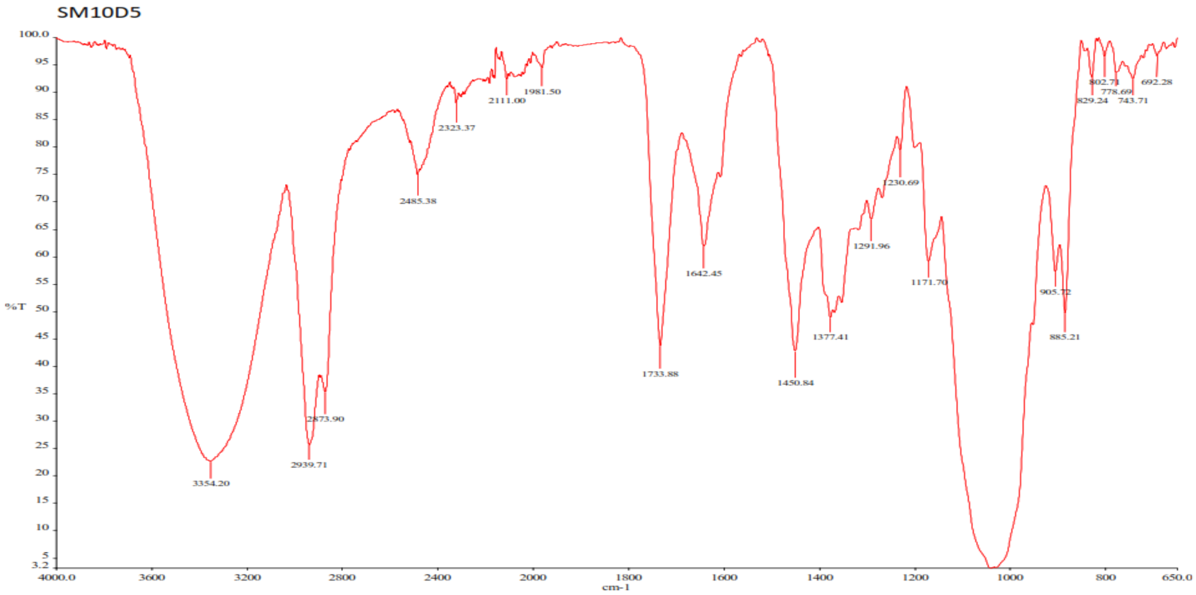

Figure S9. IR spectrum of compound **2**

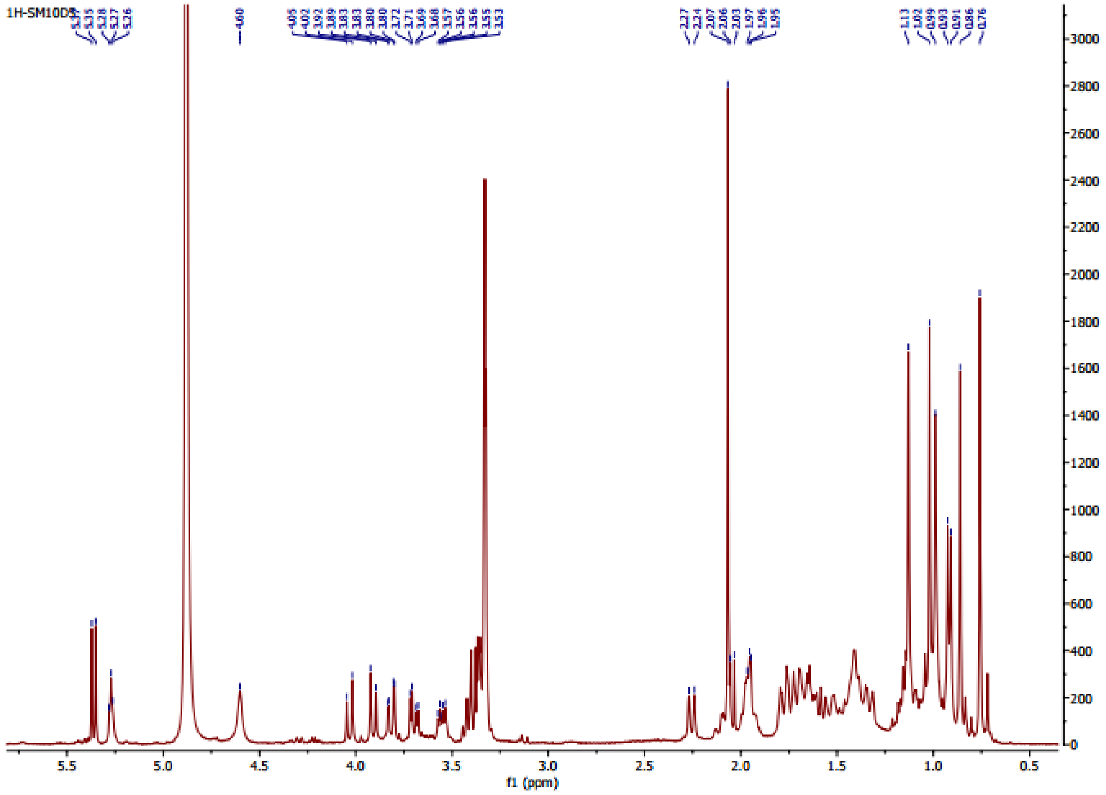

Figure S10. ^{1}H NMR spectrum of compound **2**

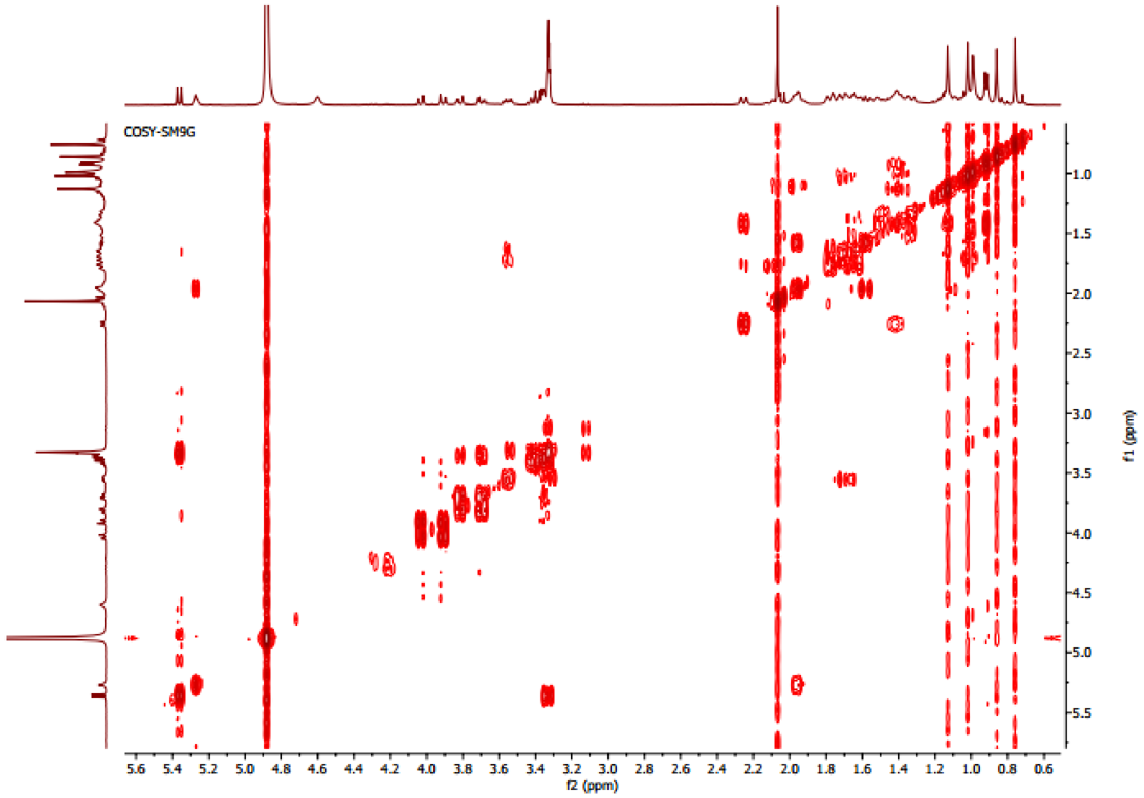

Figure S11. ^{1}H-^{1}H COSY spectrum of compound **2**

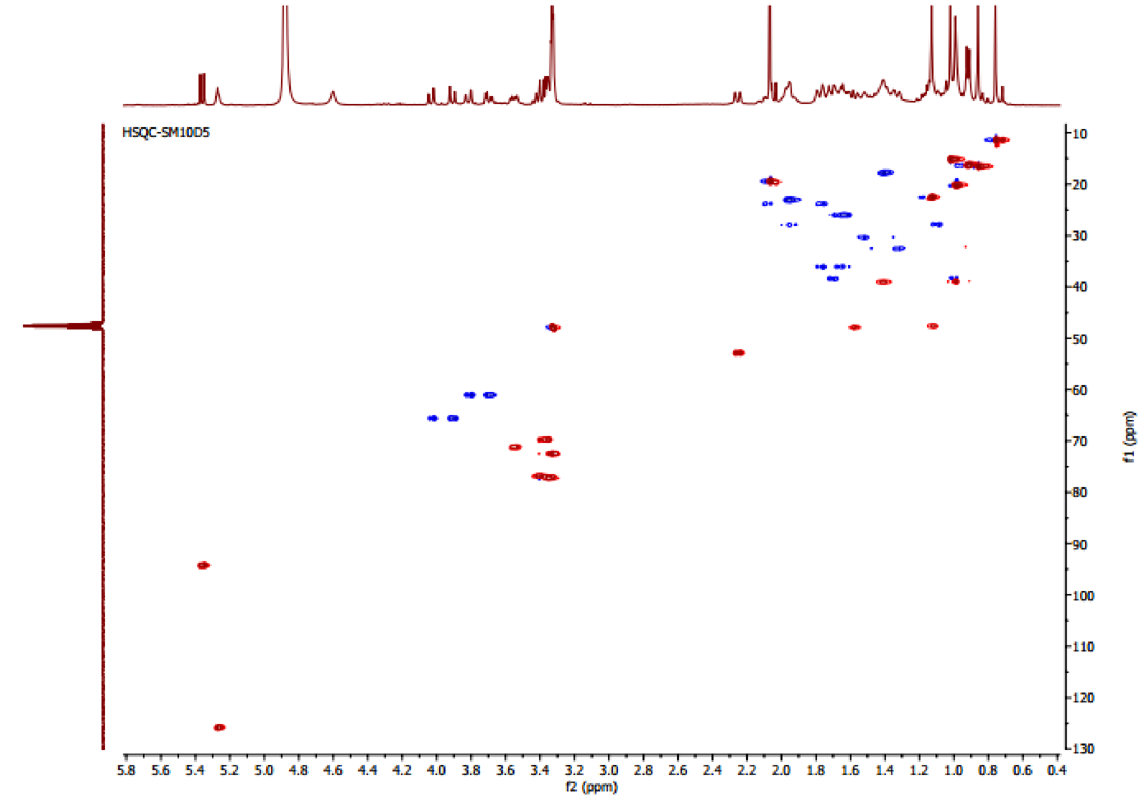

Figure S12. HSQC spectrum of compound **2**

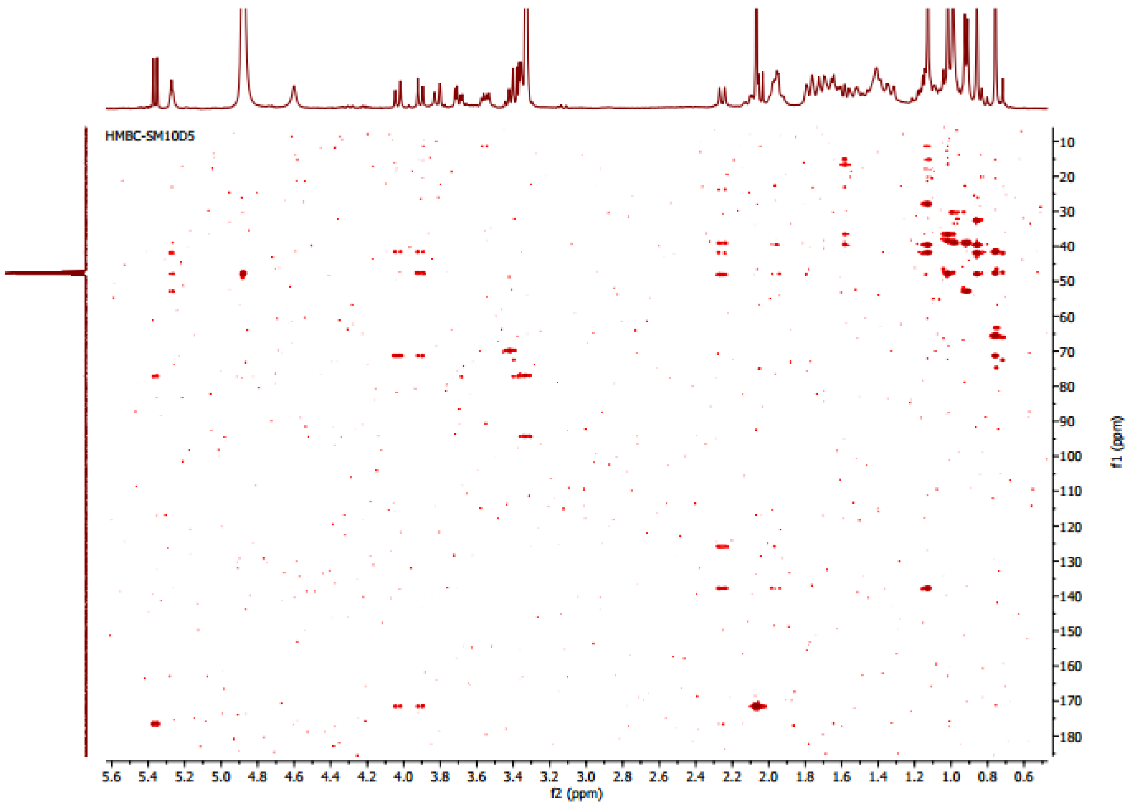

Figure S13. HMBC spectrum of compound **2**

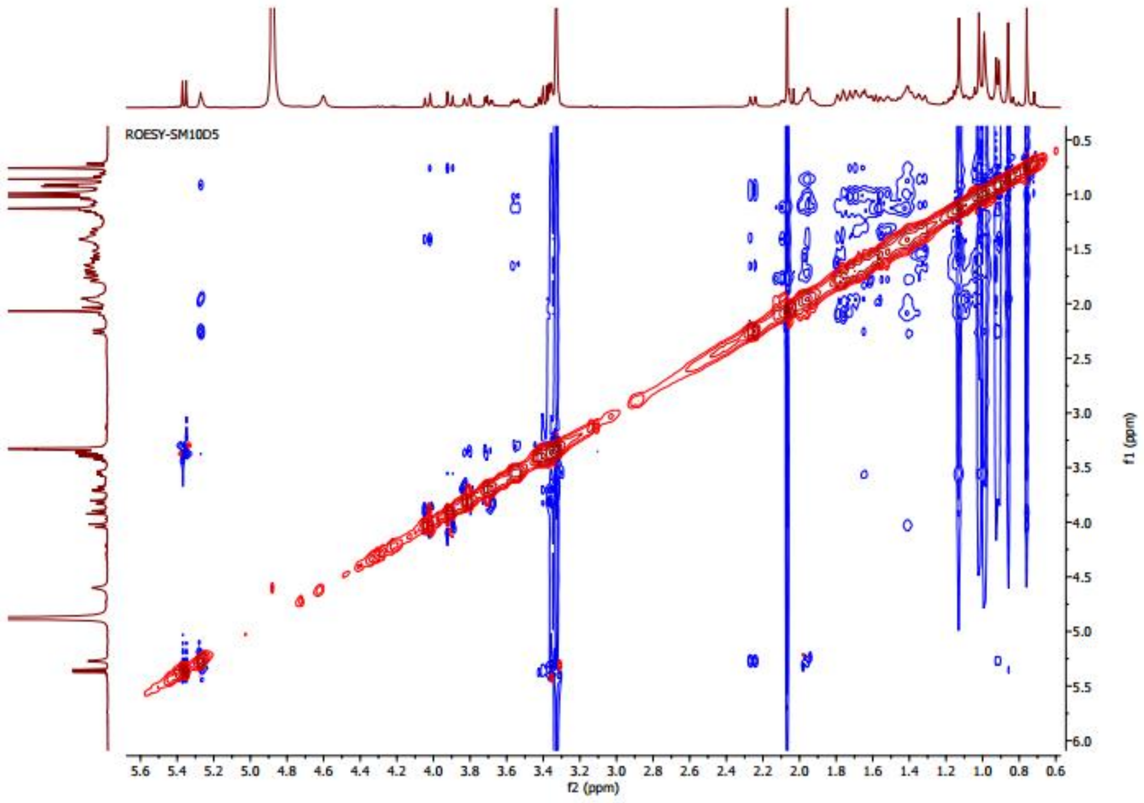

Figure S14. ROESY spectrum of compound **2**

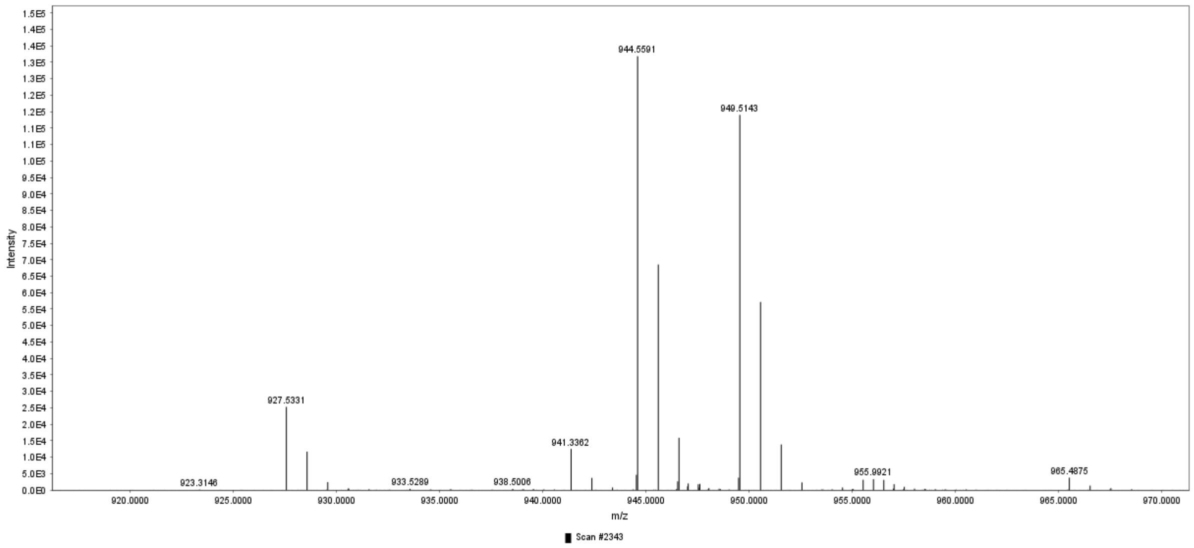

Figure S15. HRESIMS of compound **3**

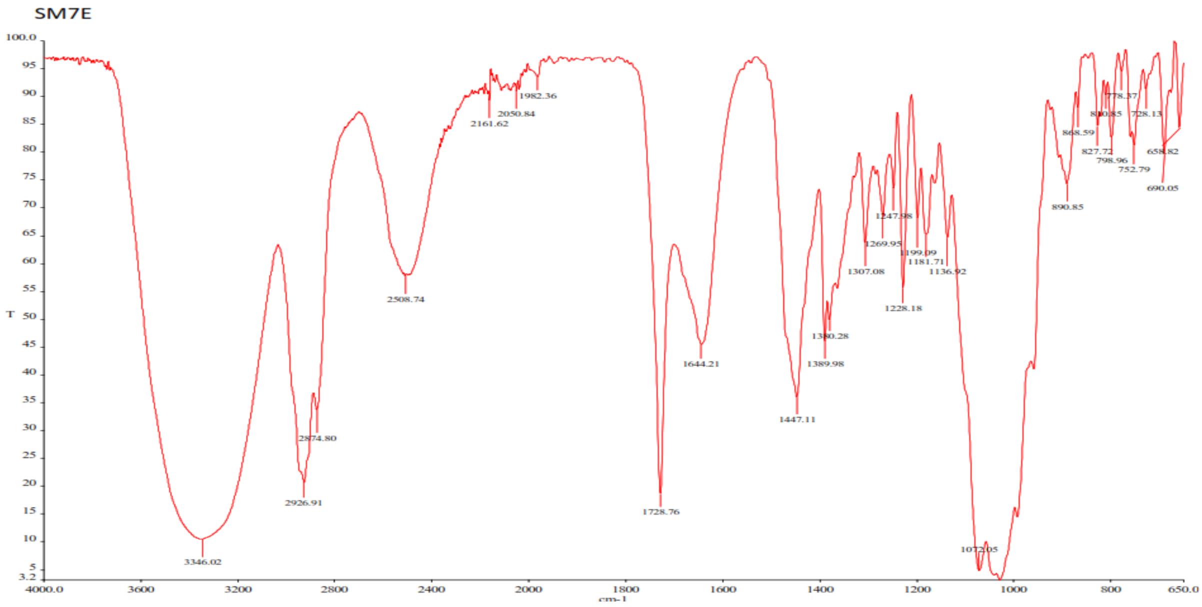

Figure S16. IR spectrum of compound **3**

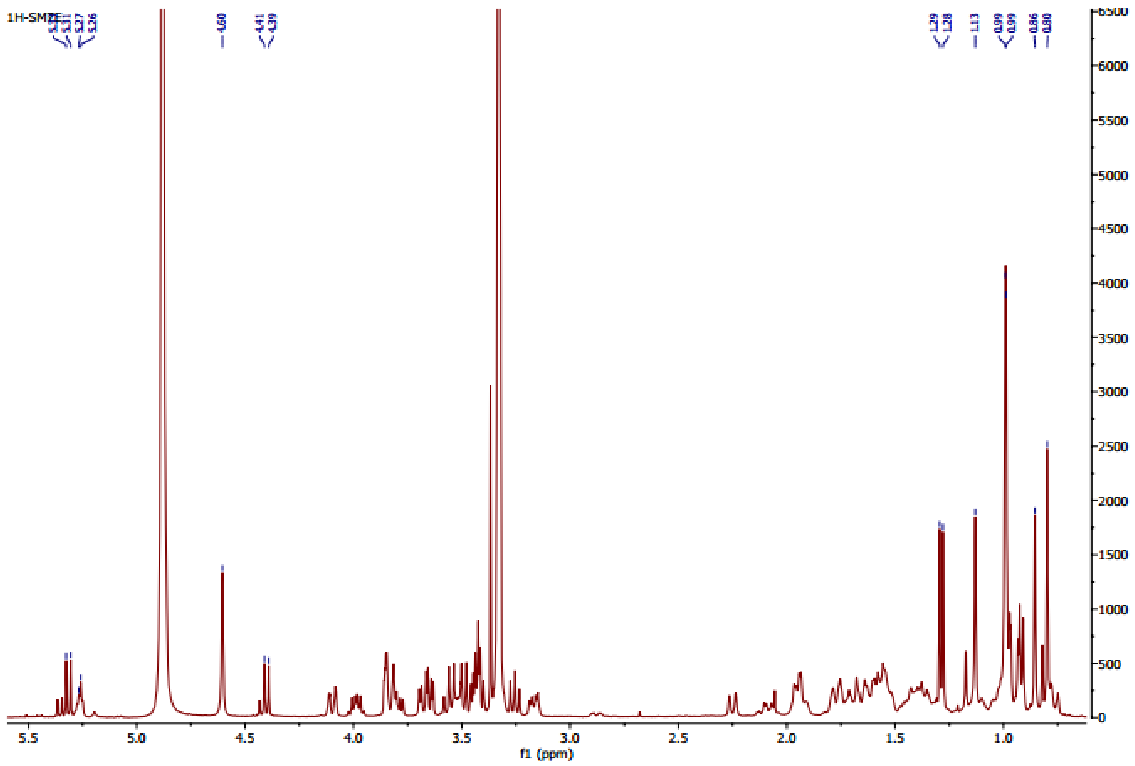

Figure S17. ^{1}H NMR spectrum of compound **3**

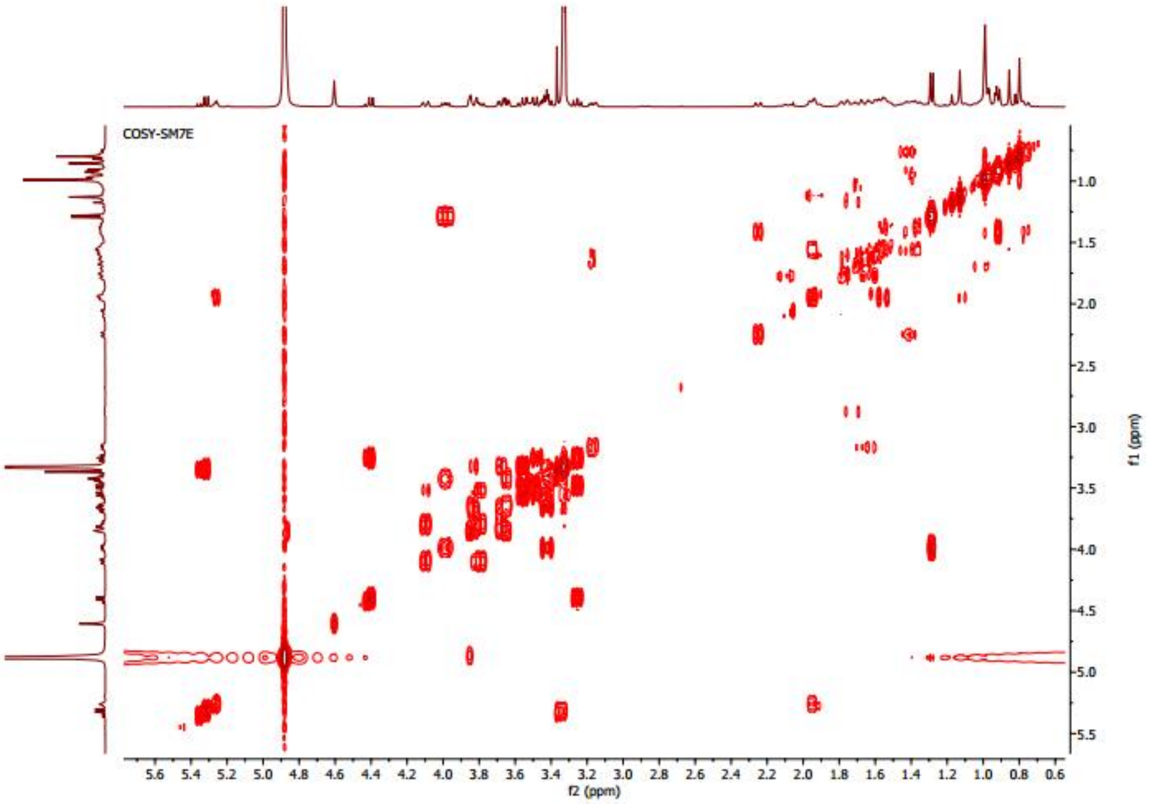

Figure S18. ^{1}H-^{1}H COSY spectrum of compound **3**

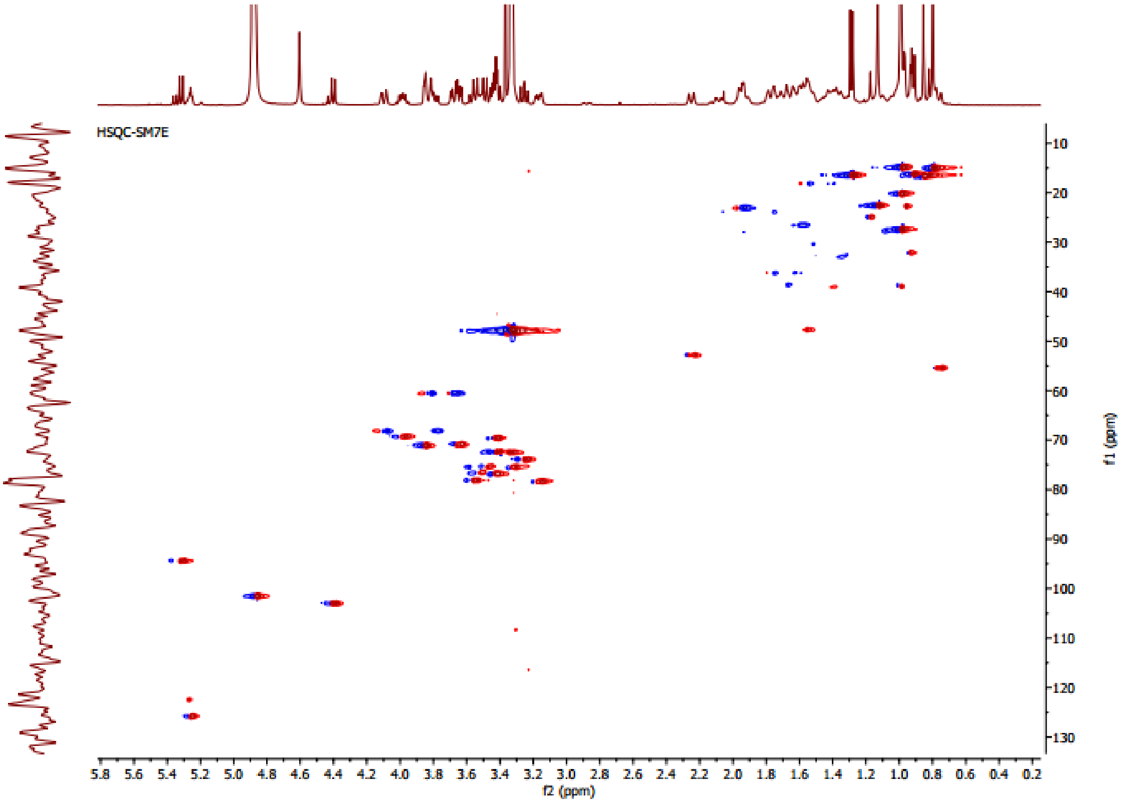

Figure S19. HSQC spectrum of compound **3**

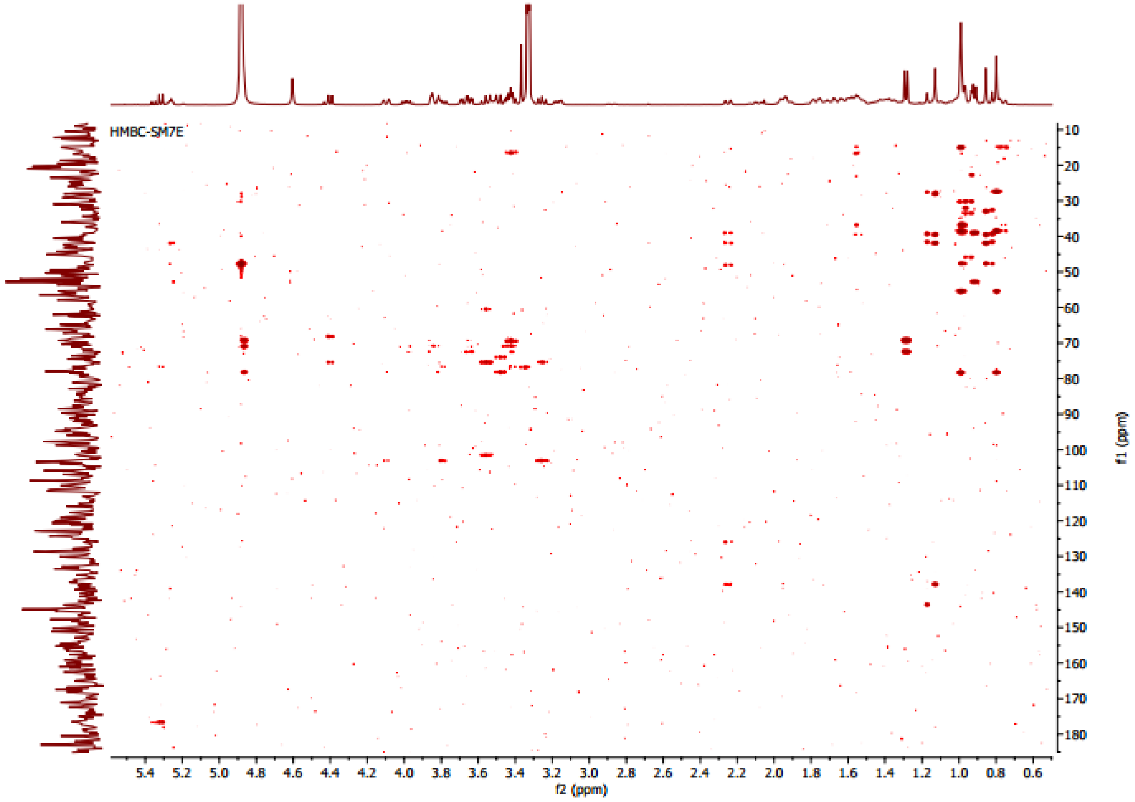

Figure S20. HMBC spectrum of compound **3**

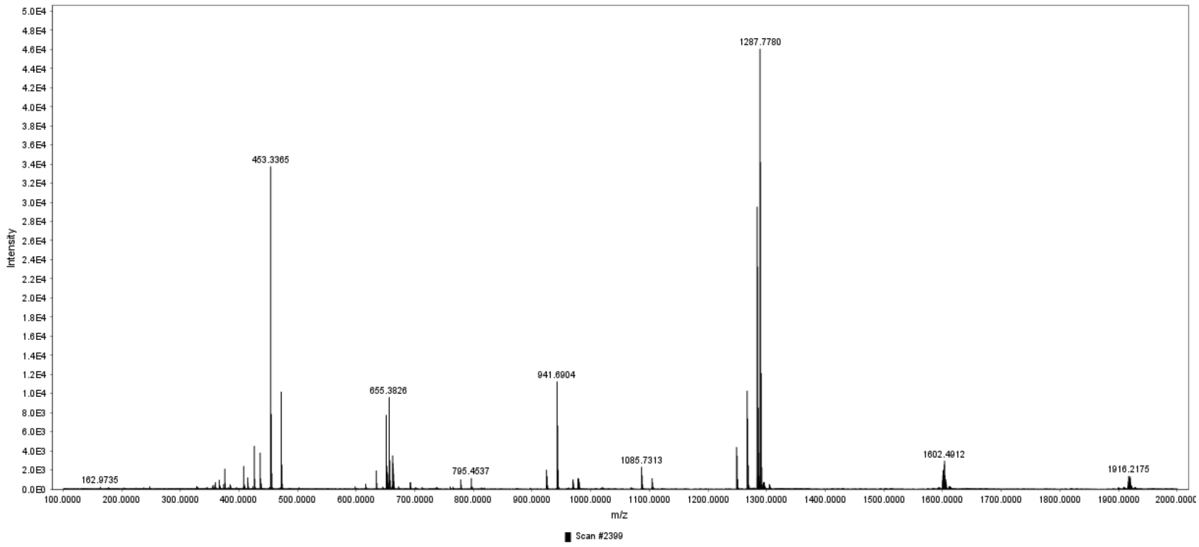

Figure S21. HRESIMS of compound **4**

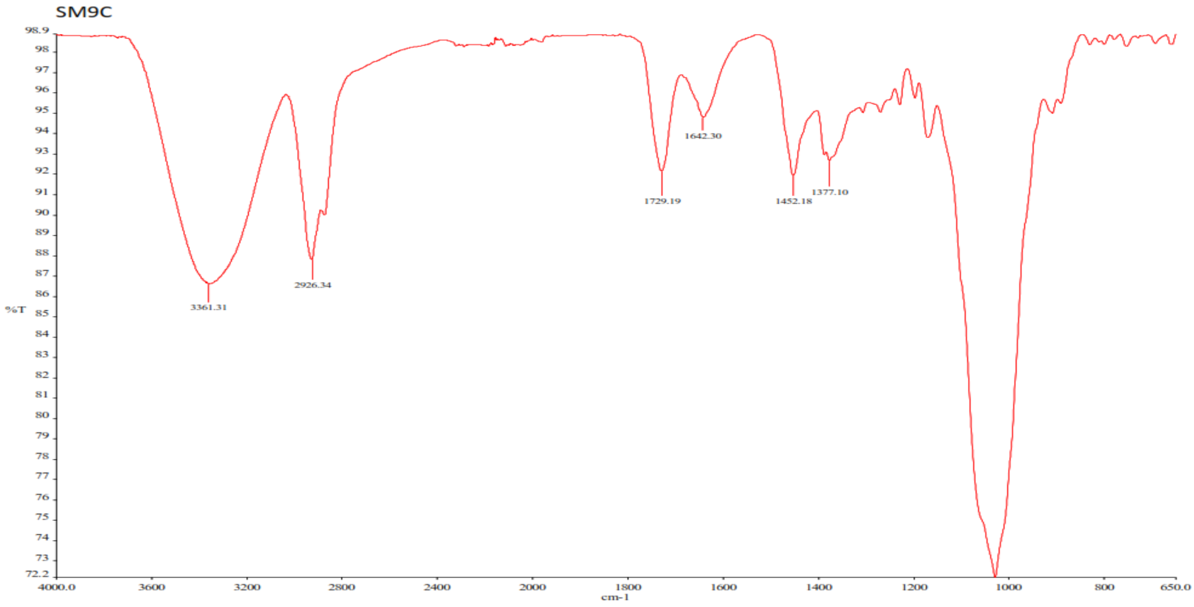

Figure S22. IR spectrum of compound **4**

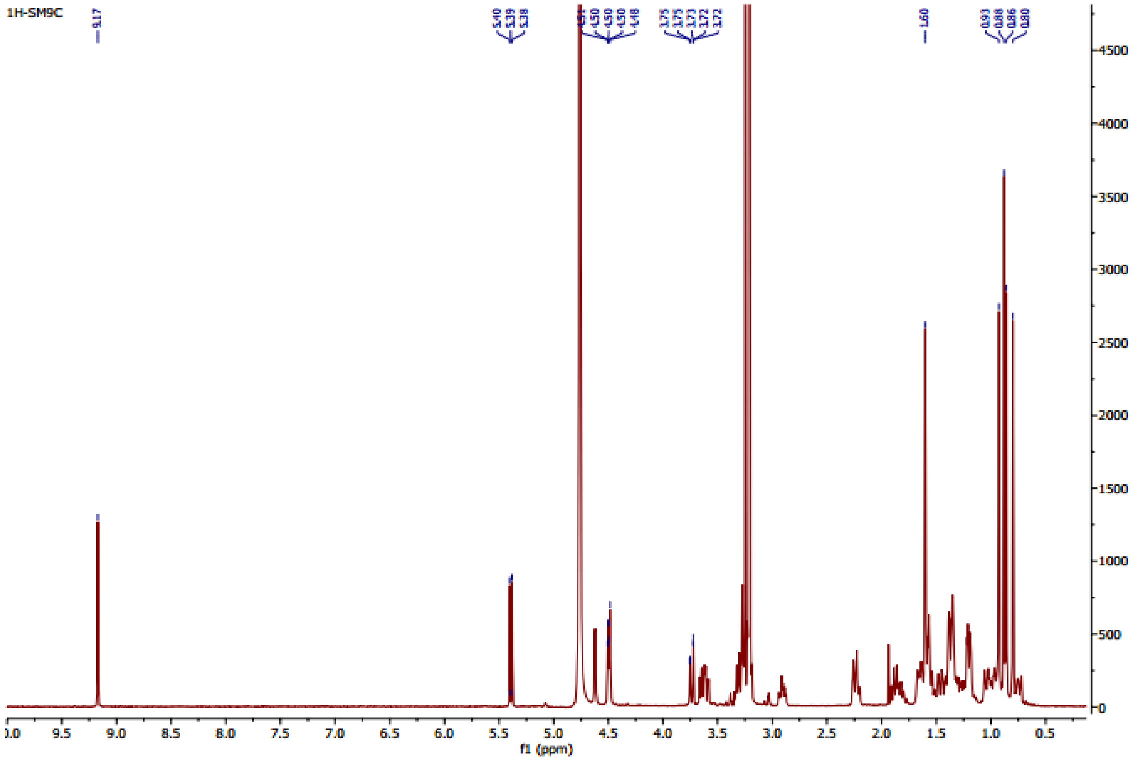

Figure S23. ^{1}H NMR spectrum of compound **4**

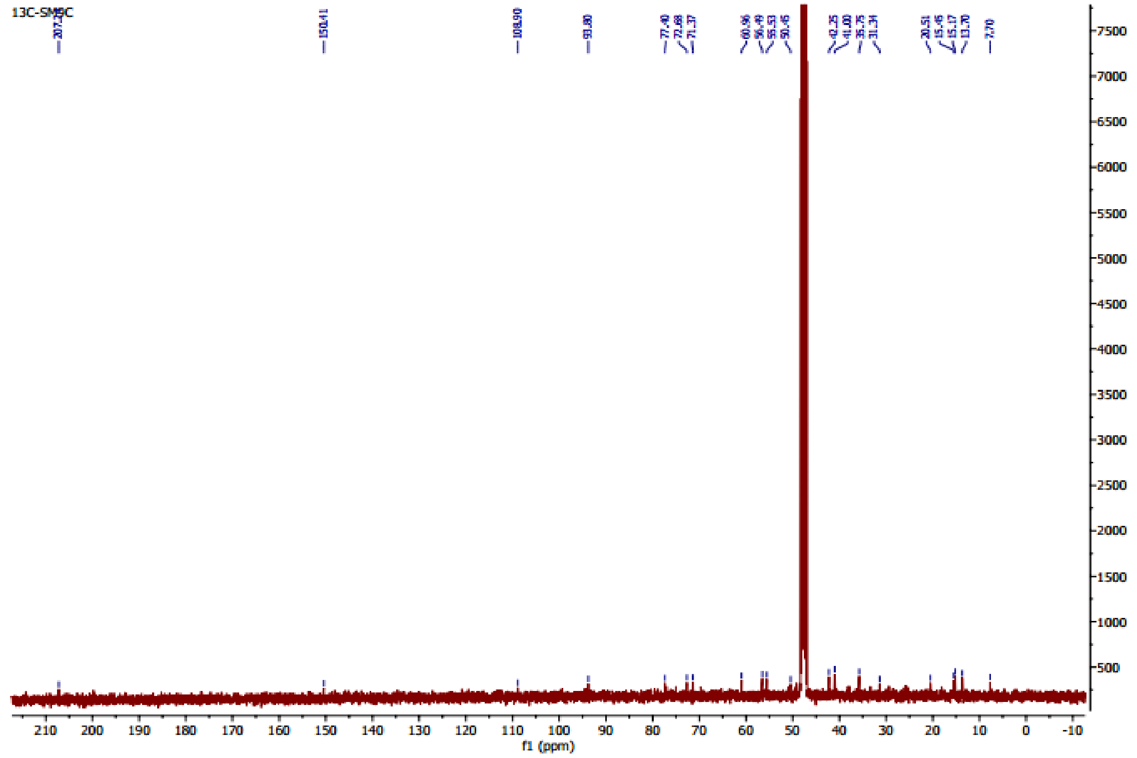

Figure S24. ^{13}C NMR spectrum of compound **4**

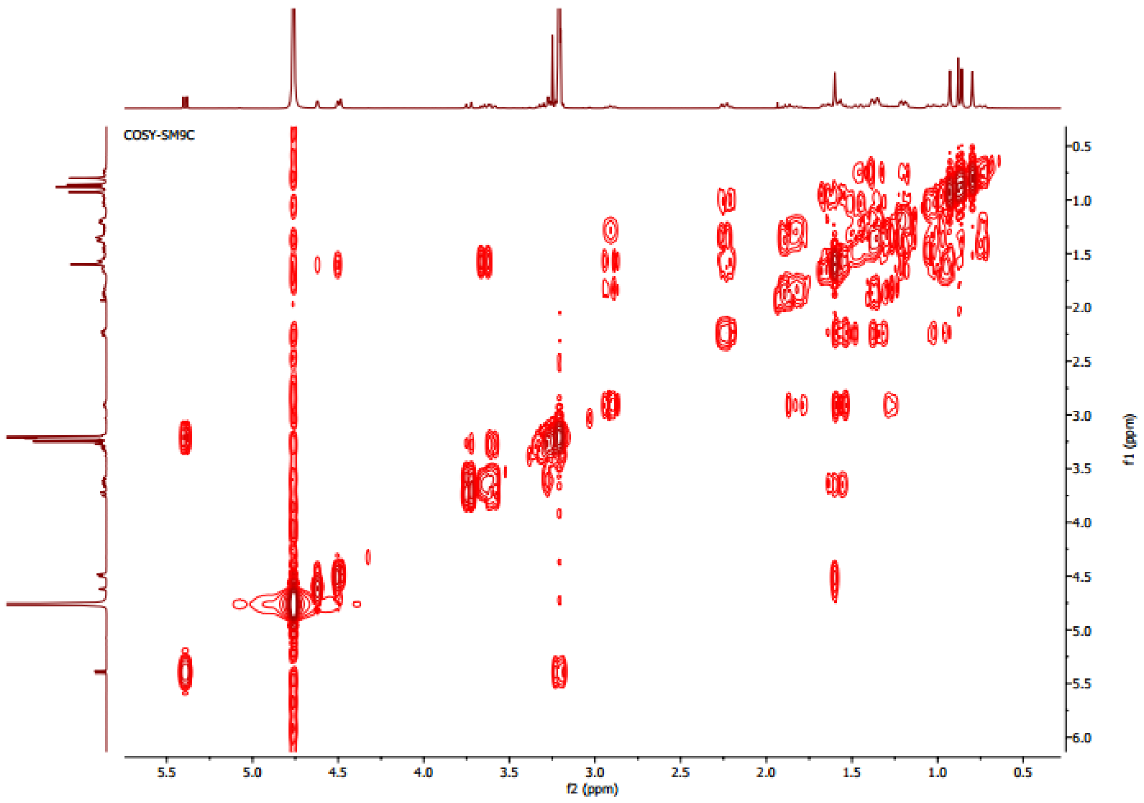

Figure S25. ^{1}H-^{1}H COSY spectrum of compound **4**

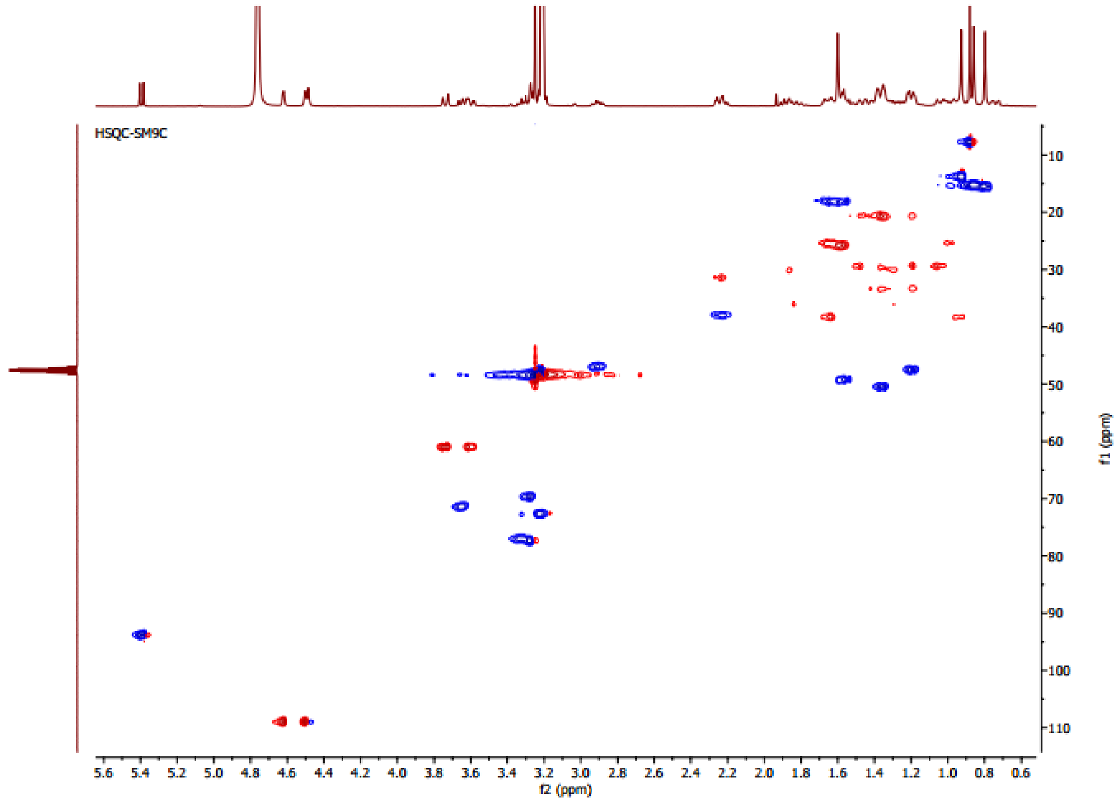

Figure S26. HSQC spectrum of compound **4**

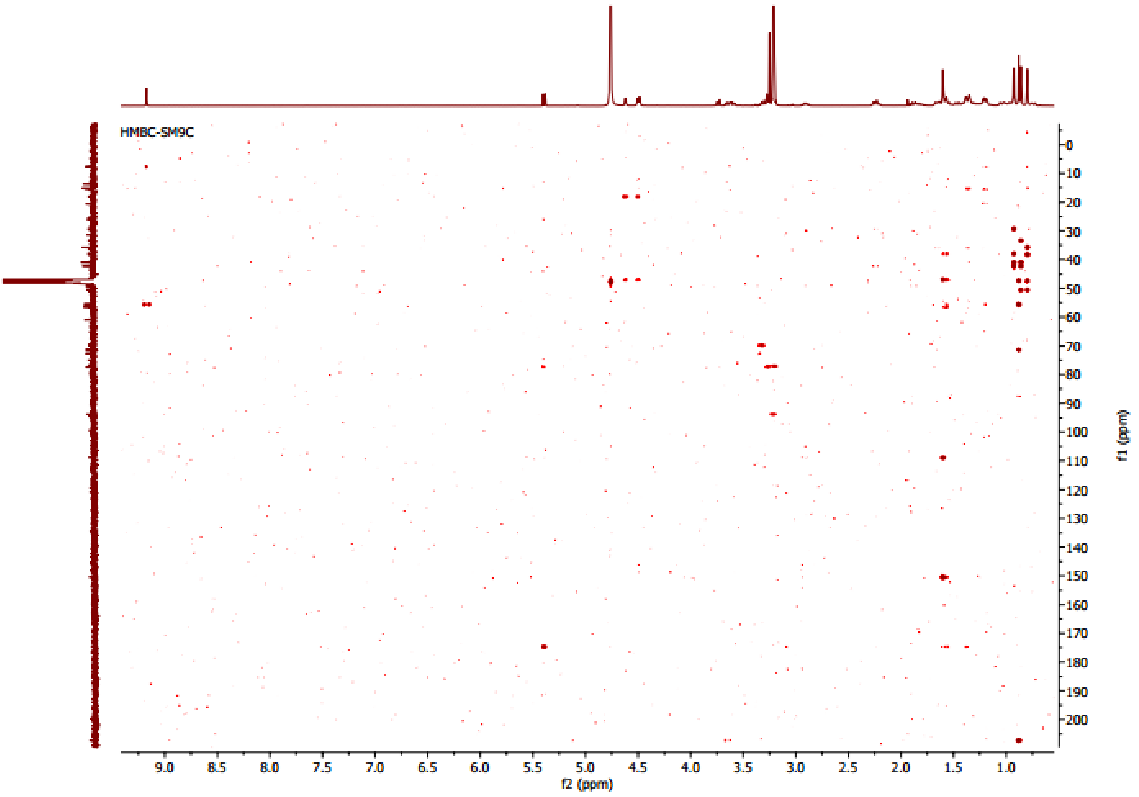

Figure S27. HMBC spectrum of compound **4**

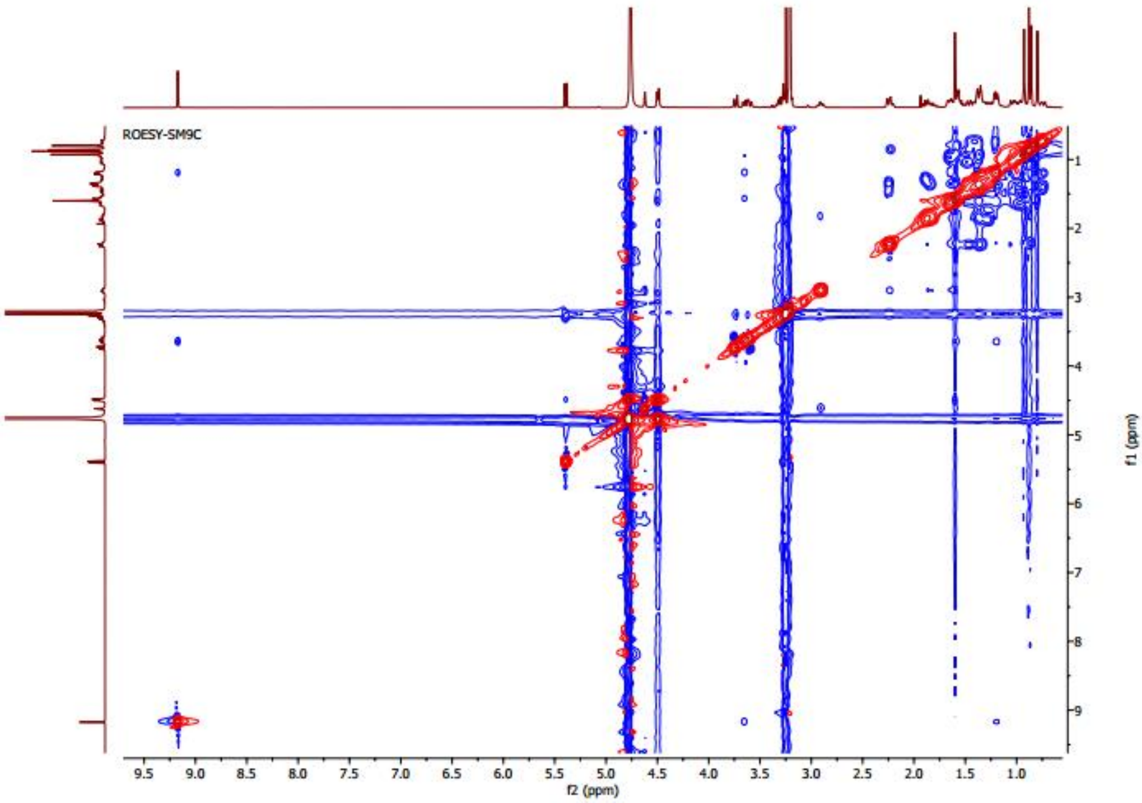

Figure S28. ROESY spectrum of compound **4**
